# Supplementary material for: Non-canonical RNA-DNA differences and other human genomic features are enriched within very short tandem repeats
Source: PLoS Comput Biol. 2020 Jun 8;16(6):e1007968. doi: 10.1371/journal.pcbi.1007968 (PMC7302867; doi:10.1371/journal.pcbi.1007968)
Supplement: S1 Table — *TNR species are designated with lowercase strings just to imply that the written TNR string actually accommodates multiple TNR motifs. For instance, TNR aat accommodates six repeat motifs: AAT, ATA, TAA, ATT, TAT, and TTA. This phenomenon is different from that of MNR and DNR, where the species name uniquely identifies a repeat motif. (DOCX) [file pcbi.1007968.s001.docx]

# Table S1. Comparative summary statistics of polytracts between GRCh38 and GRCh37.

|  | GRCh38 | | | | GRCh37 | | | | |
| --- | --- | --- | --- | --- | --- | --- | --- | --- | --- |
| **Polytract*** | **# tracts** | **Tract volume (nt)** | | **Genome occupancy** | **# tracts** | **Tract volume (nt)** | | **Genome occupancy** | |
| A/T | 7,119,220 | 55,290,931 | 1.79% | | 7,061,825 | | 54,891,880 | | 1.77% |
| C/G | 610,474 | 3,839,875 | 0.12% | | 606,810 | | 3,816,629 | | 0.12% |
| *MNR_total* | *7,729,694* | *59,130,806* | *1.91%* | | *7,668,635* | | *58,708,509* | | 1.90% |
| TA | 2,764,278 | 19,948,282 | 0.65% | | 2,734,627 | | 19,743,803 | | 0.64% |
| CT/GA | 3,679,922 | 25,030,351 | 0.81% | | 3,592,222 | | 24,446,671 | | 0.79% |
| CA/GT | 3,371,036 | 25,125,048 | 0.81% | | 3,150,860 | | 23,647,582 | | 0.76% |
| GC | 71,160 | 476,554 | 0.02% | | 70,428 | | 471,668 | | 0.02% |
| *DNR_total* | *9,886,396* | *70,580,235* | *2.29%* | | *9,548,137* | | *68,309,724* | | 2.21% |
| aat | 353,551 | 3,828,512 | 0.12% | | 353,125 | | 3,815,091 | | 0.12% |
| acc | 238,068 | 2,302,369 | 0.07% | | 236,953 | | 2,290,504 | | 0.07% |
| aag | 183,579 | 1,859,914 | 0.06% | | 182,507 | | 1,848,670 | | 0.06% |
| agg | 183,232 | 1,856,718 | 0.06% | | 182,151 | | 1,845,877 | | 0.06% |
| aac | 146,444 | 1,720,479 | 0.06% | | 145,749 | | 1,712,281 | | 0.06% |
| cag | 131,235 | 1,290,213 | 0.04% | | 130,507 | | 1,282,879 | | 0.04% |
| atc | 123,051 | 1,223,622 | 0.04% | | 114,228 | | 1,140,539 | | 0.04% |
| act | 36,083 | 352,774 | 0.01% | | 35,983 | | 351,696 | | 0.01% |
| cgg | 21,508 | 238,039 | 0.01% | | 20,857 | | 231,159 | | 0.01% |
| gac | 1,396 | 13,915 | 0.00% | | 1,382 | | 13,743 | | 0.00% |
| *TNR_total* | *1,418,147* | *14,686,555* | *0.48%* | | *1,403,442* | | *14,532,439* | | 0.47% |

*TNR species are designated with lowercase strings just to imply that the written TNR string actually accommodates multiple TNR motifs. For instance, TNR aat accommodates six repeat motifs: AAT, ATA, TAA, ATT, TAT, and TTA. This phenomenon is different from that of MNR and DNR, where the species name uniquely identifies a repeat motif.
