# Supplementary material for: Non-canonical RNA-DNA differences and other human genomic features are enriched within very short tandem repeats
Source: PLoS Comput Biol. 2020 Jun 8;16(6):e1007968. doi: 10.1371/journal.pcbi.1007968 (PMC7302867; doi:10.1371/journal.pcbi.1007968)
Supplement: S3 Table — (DOCX) [file pcbi.1007968.s003.docx]

# Table S3. Genomic features covered in polytract feature enrichment landscapes.

| Feature category | Feature name | Feature Name in Figure5 | # records |
| --- | --- | --- | --- |
| RNA-binding protein's binding segments | FMR1 | FMR1_bound | 2,417 |
|  | FXR1 | FXR1_bound | 2,413 |
|  | FXR2 | FXR2_bound | 2,172 |
|  | HNRNPA1 | HNRNPA1_bound | 2,043 |
|  | HNRNPC | HNRNPC_bound | 2,434 |
|  | IGF2BP1 | IGF2BP1_bound | 5,265 |
|  | IGF2BP2 | IGF2BP2_bound | 4,339 |
|  | IGF2BP3 | IGF2BP3_bound | 4,133 |
|  | KHDRBS1 | KHDRBS1_bound | 2,735 |
|  | LIN28B | LIN28B_bound | 4,298 |
|  | NONO | NONO_bound | 3,510 |
|  | PCBP2 | PCBP2_bound | 3,300 |
|  | PTBP1 | PTBP1_bound | 2,282 |
|  | PUM2 | PUM2_bound | 3,715 |
|  | QKI | QKI_bound | 1,620 |
|  | RBFOX2 | RBFOX2_bound | 2,301 |
|  | RBM5 | RBM5_bound | 3,838 |
|  | SF3B4 | SF3B4_bound | 1,879 |
|  | SFPQ | SFPQ_bound | 758 |
|  | SRSF1 | SRSF1_bound | 3,617 |
|  | SRSF7 | SRSF7_bound | 2,181 |
|  | SRSF9 | SRSF9_bound | 3,123 |
|  | TAF15 | TAF15_bound | 1,565 |
|  | TARDBP | TARDBP_bound | 2,438 |
|  | TIA1 | TIA1_bound | 5,755 |
|  | U2AF2 | U2AF2_bound | 2,233 |
| enhancer | UE | uEnhancer | 123 |
|  | nsEnhancer | nsEnhancer | 1,134 |
|  | sEnhancer | sEnhancer | 4,833 |
|  | enhancers | enhancers | 5,967 |
| miscellaneous | eQTL | eQTL | 2,611,419 |
|  | blacklist | blacklist | 167,144 |
|  | gnomAD_indel | gnomAD_indel | 66,396,448 |
|  | gnomAD_SNV | gnomAD_SNV | 227,172,514 |
|  | H3K27ac | H3K27ac | 1,047 |
|  | H3K27me3 | H3K27me3 | 582 |
|  | H3K36me3 | H3K36me3 | 2,310 |
|  | H3K4me1 | H3K4me1 | 1,824 |
|  | H3K4me3 | H3K4me3 | 4,085 |
|  | H3K9me3 | H3K9me3 | 626 |
|  | LINE_1 | LINE_1 | 253 |
|  | RIP | RIP | 17,302 |
|  | TFBS | TFBS | 5,490,818 |
|  | TCGAmut_indel | TCGAmut_indel | 165,049 |
|  | TCGAmut_SNV | TCGAmut_SNV | 2,618,017 |
| Gene regions in HG38 | 3prime_overlapping_ncRNA | 3o_ncRNA | 78 |
|  | antisense | antisense | 25,196 |
|  | bidirectional_promoter_lncRNA | bp_lncRNA | 622 |
|  | IG_C_gene | IG_C_gene | 143 |
|  | IG_C_pseudogene | IG_C_pseudogene | 16 |
|  | IG_D_gene | IG_D_gene | 44 |
|  | IG_J_gene | IG_J_gene | 25 |
|  | IG_V_gene | IG_V_gene | 694 |
|  | IG_V_pseudogene | IG_V_pseudogene | 289 |
|  | lincRNA | lincRNA | 34,364 |
|  | macro_lncRNA | macro_lncRNA | 2 |
|  | miRNA | miRNA | 1,867 |
|  | misc_RNA | misc_RNA | 2,222 |
|  | non_coding | non_coding | 3 |
|  | polymorphic_pseudogene | p_pseudogene | 581 |
|  | processed_pseudogene | P_PSEUDOGENE | 11,821 |
|  | processed_transcript | p_transcript | 8,467 |
|  | protein_coding | protein_coding | 734,549 |
|  | pseudogene | pseudogene | 49 |
|  | ribozyme | ribozyme | 9 |
|  | rRNA | rRNA | 52 |
|  | rRNA_pseudogene | rRNA_pseudogene | 498 |
|  | scaRNA | scaRNA | 50 |
|  | scRNA | scRNA | 2 |
|  | sense_intronic | sense_intronic | 1,690 |
|  | sense_overlapping | s_overlapping | 721 |
|  | snoRNA | snoRNA | 956 |
|  | snRNA | snRNA | 1,910 |
|  | sRNA | sRNA | 6 |
|  | TEC | TEC | 1,100 |
|  | transcribed_processed_pseudogene | tp_pseudogene | 1,987 |
|  | transcribed_unitary_pseudogene | tu_pseudogene | 2,313 |
|  | transcribed_unprocessed_pseudogene | TU_PSEUDOGENE | 10,743 |
|  | translated_processed_pseudogene | TP_PSEUDOGENE | 4 |
|  | TR_C_gene | TR_C_gene | 42 |
|  | TR_D_gene | TR_D_gene | 5 |
|  | TR_J_gene | TR_J_gene | 80 |
|  | TR_V_gene | TR_V_gene | 486 |
|  | TR_V_pseudogene | TR_V_pseudogene | 58 |
|  | unitary_pseudogene | u_pseudogene | 293 |
|  | unprocessed_pseudogene | U_PSEUDOGENE | 8,049 |
| RNA-DNA Difference | a2c | a2c_RDD | 1,158 |
|  | A2G | A2G_RDD | 4,677,846 |
|  | a2t | a2t_RDD | 1,649 |
|  | C2T | C2T_RDD | 5,006 |
|  | g2c | g2c_RDD | 700 |
|  | g2t | g2t_RDD | 2,136 |
|  | non-A2G | non-A2G_RDD | 10,649 |
|  | non-canonical | nc_RDD | 5,643 |
