# Supplementary material for: Non-canonical RNA-DNA differences and other human genomic features are enriched within very short tandem repeats
Source: PLoS Comput Biol. 2020 Jun 8;16(6):e1007968. doi: 10.1371/journal.pcbi.1007968 (PMC7302867; doi:10.1371/journal.pcbi.1007968)
Supplement: S4 Table — (DOCX) [file pcbi.1007968.s004.docx]

# Table S4. Comparison of Polytrap against related tools

| **Tool** | **Origination** | **STR-relevant capability** | **Object in overlap** | **Overlap statistical test** | **manageable organisms** |
| --- | --- | --- | --- | --- | --- |
| Polytrap | the present work | Spatial overlap with STR | {custom intervals} against {STRs of MNR/DNR/TNR motif} | Binomial test | human, macaque, mouse, rat, dot, chicken, fugu, fruitfly, and yeast |
| GLANET [^1^](#_ENREF_1) | Otlu et al., 2017 (Bioinformatics) | N/A | {custom intervals} against {non-coding regulatory elements and gene-centric elements} | Sampling-based approach, z score | human |
| GREAT [^2^](#_ENREF_2) | McLean et al., 2010 (Nature Biotechnology) | N/A | {custom intervals} against {well-characterized genes} | Binomial test, Hypergeometric test | human, mouse, zebrafish |
| ANNOVAR [^3^](#_ENREF_3) | Wang et al., 2010 (Nucleic Acids Research) | N/A | {custom intervals} against {non-coding regulatory elements and gene-centric elements} | N/A | human and common model organisms |
| Krait [^4^](#_ENREF_4) | Du et al., 2018 (Bioinformatics) | STR discovery | {ab-initio detected STRs} against {gene-centric elements} | N/A | Any represented in genome-wide FASTA sequence(s) |
| WGSSAT [^5^](#_ENREF_5) | Pandey et al., 2018 (J Hered) | STR discovery | {ab-initio detected STRs} against {gene-centric elements} | N/A | Any represented in genome-wide FASTA sequence(s) |
| Dot2dot [^6^](#_ENREF_6) | Genovese et al., 2019 (Bioinformatics) | STR discovery | N/A | N/A | Any represented in genome-wide FASTA sequence(s) |
| lobSTR [^7^](#_ENREF_7) | Gymrek et al., 2012 (Genome Research) | STR profiling | N/A | N/A | human |
| STRScan [^8^](#_ENREF_8) | Tang & Nzabarushimana, 2017 (BMC Bioinformatics) | STR profiling | N/A | N/A | human |
| popSTR [^9^](#_ENREF_9) | Kristmundsdóttir et al., 2016 (Bioinformatics) | STR profiling | N/A | N/A | human |
| Dante [^10^](#_ENREF_10) | Budis et al., 2019 (Bioinformatics) | STR profiling | N/A | N/A | human |

# Reference

1. Otlu, B., Firtina, C., Keles, S. & Tastan, O. GLANET: genomic loci annotation and enrichment tool. *Bioinformatics* **33**, 2818-2828 (2017).

2. McLean, C.Y. *et al.* GREAT improves functional interpretation of cis-regulatory regions. *Nat Biotechnol* **28**, 495-501 (2010).

3. Wang, K., Li, M. & Hakonarson, H. ANNOVAR: functional annotation of genetic variants from high-throughput sequencing data. *Nucleic Acids Res* **38**, e164 (2010).

4. Du, L. *et al.* Krait: an ultrafast tool for genome-wide survey of microsatellites and primer design. *Bioinformatics* **34**, 681-683 (2018).

5. Pandey, M. *et al.* WGSSAT: A High-Throughput Computational Pipeline for Mining and Annotation of SSR Markers From Whole Genomes. *J Hered* **109**, 339-343 (2018).

6. Genovese, L.M. *et al.* A Census of Tandemly Repeated Polymorphic Loci in Genic Regions Through the Comparative Integration of Human Genome Assemblies. *Front Genet* **9**, 155 (2018).

7. Gymrek, M., Golan, D., Rosset, S. & Erlich, Y. lobSTR: A short tandem repeat profiler for personal genomes. *Genome Res* **22**, 1154-62 (2012).

8. Tang, H. & Nzabarushimana, E. STRScan: targeted profiling of short tandem repeats in whole-genome sequencing data. *BMC Bioinformatics* **18**, 398 (2017).

9. Kristmundsdottir, S., Sigurpalsdottir, B.D., Kehr, B. & Halldorsson, B.V. popSTR: population-scale detection of STR variants. *Bioinformatics* **33**, 4041-4048 (2017).

10. Budis, J. *et al.* Dante: genotyping of known complex and expanded short tandem repeats. *Bioinformatics* **35**, 1310-1317 (2019).
