# Supplementary figures and images for: Non-canonical RNA-DNA differences and other human genomic features are enriched within very short tandem repeats
Source: PLoS Comput Biol. 2020 Jun 8;16(6):e1007968. doi: 10.1371/journal.pcbi.1007968 (PMC7302867; doi:10.1371/journal.pcbi.1007968)

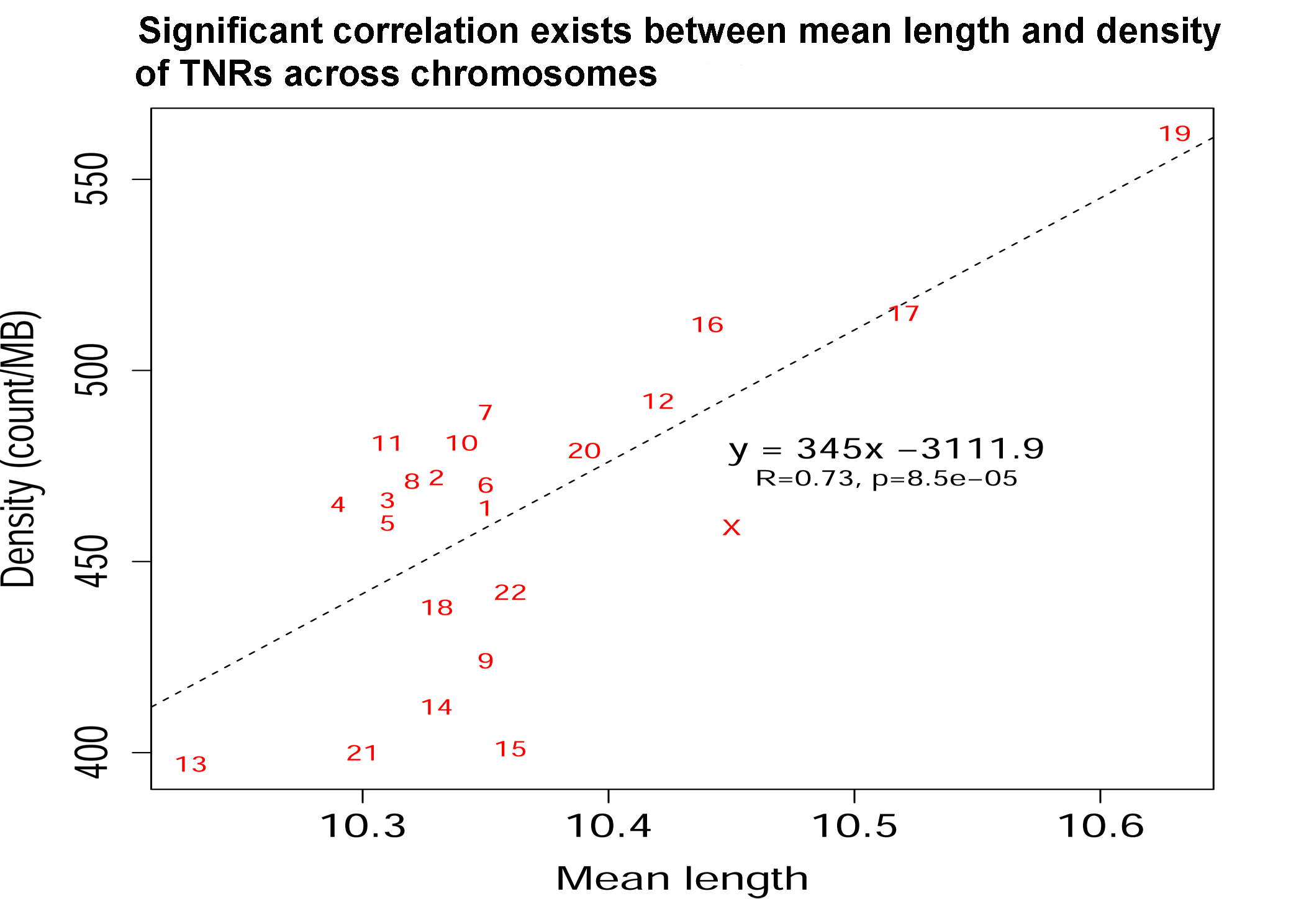

Supplement: S1 Fig — (TIF) [file pcbi.1007968.s005.tif]

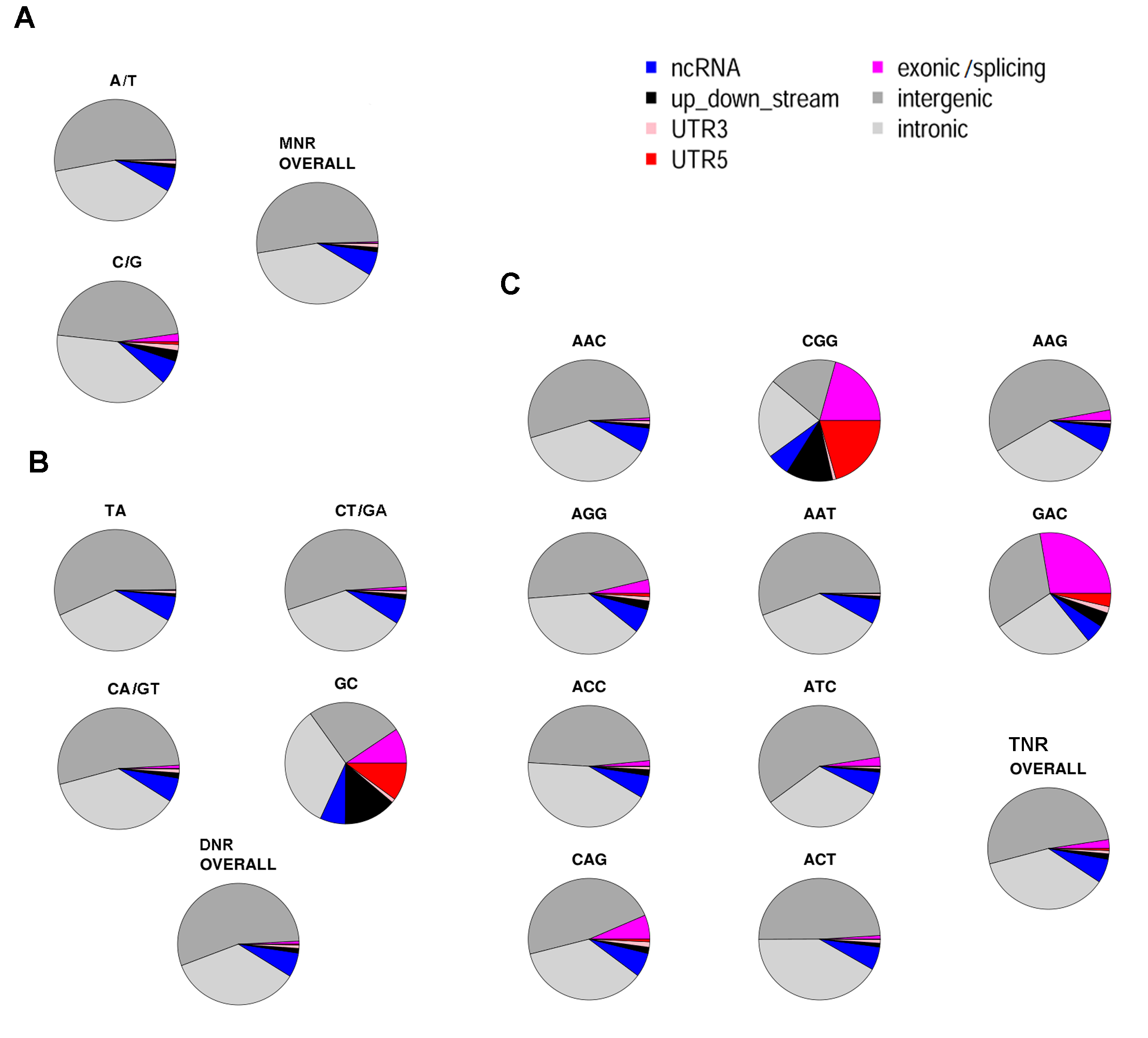

Supplement: S2 Fig — A, MNR. B, DNR. C, TNR. (TIF) [file pcbi.1007968.s006.tif]

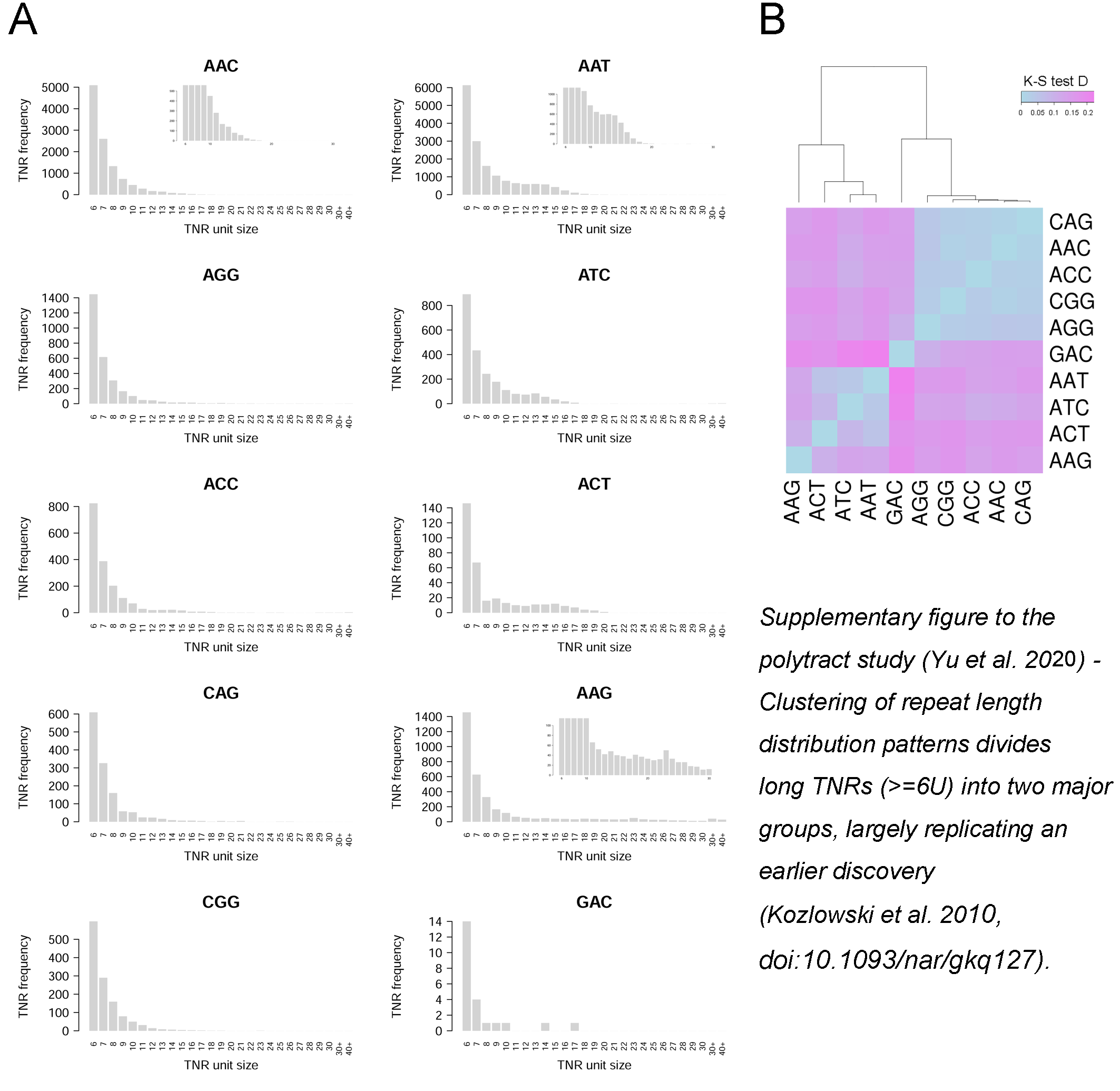

Supplement: S3 Fig — From the total 1,418,147 TNR tracts, we identified a subset of 39,937 long polytracts that each contained six or more (complete or incomplete) trimer units (i.e., length ≥ 18nt). By repeating the same precedent analytics as performed by Kozlowski et al., we revealed largely the same length distributions for the ten TNR species, and rendered a similar TNR clustering based on a matrix of pairwise Kolmogorov-Smirnov test statistics (S3 Fig). A, polytract length distribution dissected by TNR species. B, heatmap graph showing the Kolmogorov-Smirnov statistics D resulting from pairwise comparisons of polytract lengths among all 10 TNR subtypes. (TIF) [file pcbi.1007968.s007.tif]

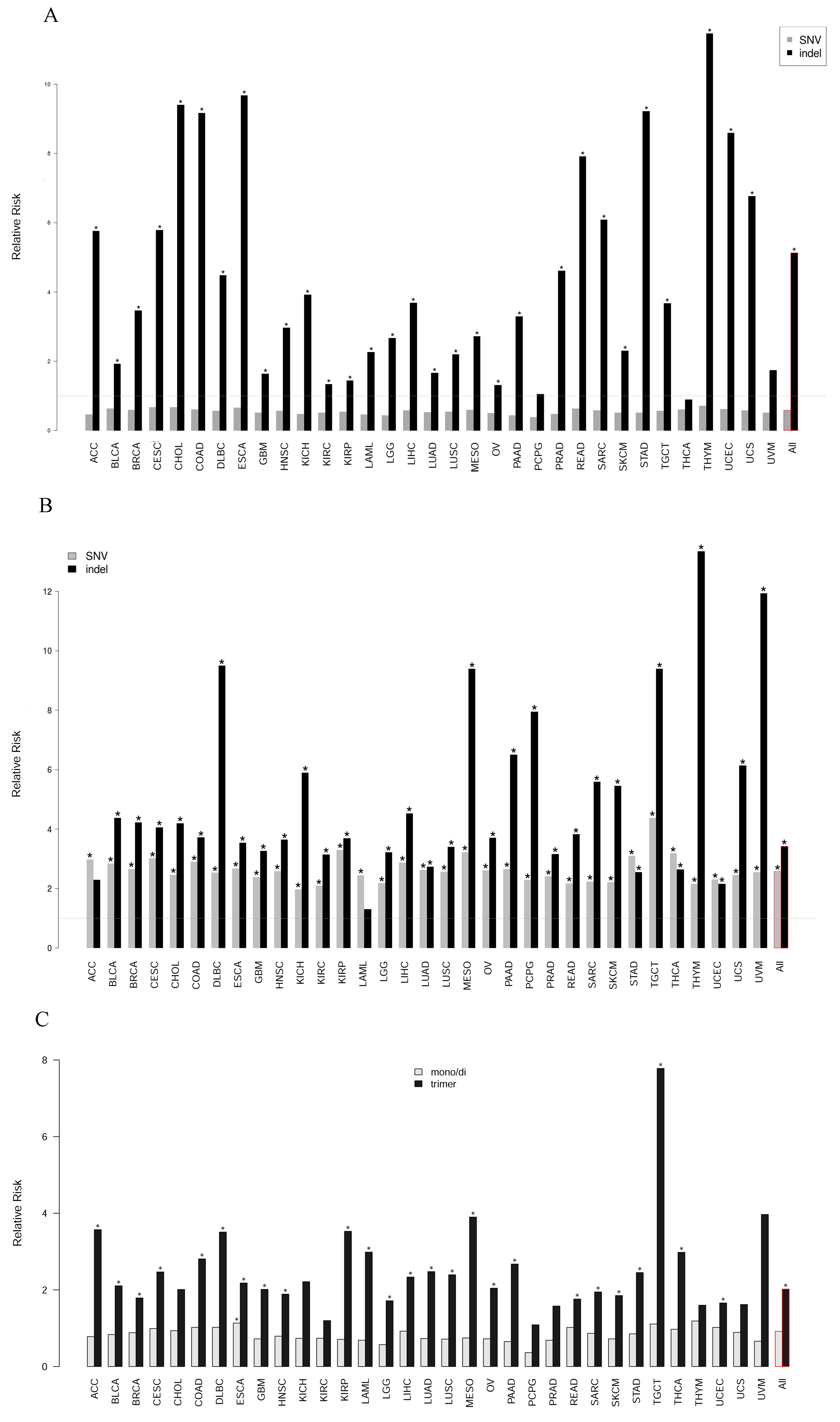

Supplement: S4 Fig — TCGA somatic SNVs were significantly over-represented within TNR polytracts but not MNR/DNR polytracts (S4 Fig, A vs. B). Because TCGA mutations were heavily biased to exomes due to experiment design and TNR polytracts have a greater exonic portion than MNR/DNR, we suspected the unique enrichment of somatic SNVs within TNR might just be a trivial reflection of the elevated exonic component of trimer tracts. To interrogate this suspect, we restricted the enrichment analysis to non-coding SNVs only, finding the qualitative distinction between MNR/DNR and TNR persisted for nearly all cancers (S4C Fig). In conclusion, we observed significant enrichment of cancer SNVs within TNR but not within MNR/DNR, even after taking into account the disparate genomic compositions of polytract clades. A, enrichment of SNVs/indels within combined monomer/dimer tracts. B, enrichment of SNVs/indels within trimer tracts. C, enrichment of non-coding SNVs within monomer/dimer or trimer tracts. Asterisk (*) indicates significant enrichment of mutations within polytract regions (p<0.01). (TIF) [file pcbi.1007968.s008.tif]

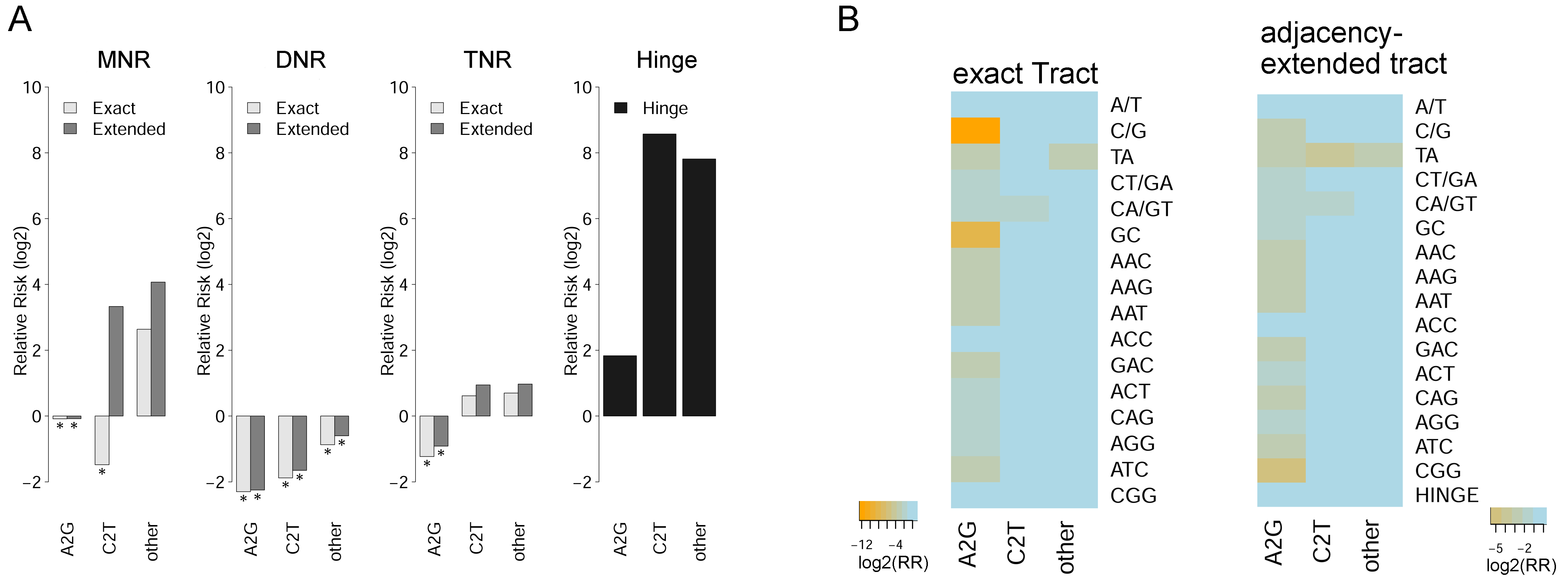

Supplement: S5 Fig — By setting the directionality option of Polytrap to “under,” we switched to investigate the under-representation tendency of three classes of RNA-editing events in polytracts, and found A-to-G events were under-represented in almost all polytract species, excluding A/T, ACC, and hinges. S5 Fig is manifested in analogous format to Fig 4, but the significance asterisk notation (*) in panel A and color shading in panel B designate under-representation rather than over-representation. A, barplot denotes extremity of Relative Risk (RR), with significant (p<0.01) under-representation tendency annotated with asterisk (*). B, heatmap of decreased RR of polytract bearing an RNA-editing event, depicted for combinations between editing event classes and polytract species. (TIF) [file pcbi.1007968.s009.tif]
