# Supplementary material for: Non-canonical RNA-DNA differences and other human genomic features are enriched within very short tandem repeats
Source: PLoS Comput Biol. 2020 Jun 8;16(6):e1007968. doi: 10.1371/journal.pcbi.1007968 (PMC7302867; doi:10.1371/journal.pcbi.1007968)
Supplement: Attachment — Submitted filename: Polytrap_Resp2referees.docx [file pcbi.1007968.s010.docx]

Hui Yu, Ph.D.

[Huiyu1@salud.unm.edu](mailto:Huiyu1@salud.unm.edu)

February 24^th^, 2020
Re: Manuscript PCOMPBIOL-D-19-01709

Dear editor,

We greatly appreciate your editorial processing and the management of our manuscript. Moreover, we are extremely grateful for the time and efforts contributed by the four referees in reviewing our manuscript. They recognized the value of our study and, more importantly, provided insightful and critical comments/suggestions. In light of these valuable feedbacks, we made efforts to improve the work and revise the manuscript in several major aspects.

Firstly, we publicized the polytract files on a stable, professional webpage, and also registered the polytract datasets of four species as official tracks in UCSC Public Tract List. Secondly, we tackled the inconsistency in treatment of reverse complement and in consequence re-defined the sub-categories of polytracts. Thirdly, we conducted a comparison between our polytracts and UCSC Microsatellites to confirm a better quality of our resultant dataset. Fourthly, we modified software Polytrap to enable the option to test for under-representation as well. In addition, we were inspired by referee’s critical opinions to thoroughly revise the manuscript, especially the Discussion and Methods part. We invite the referees to inspect our point-to-point responses below as well as the revisions in the main document that were highlighted in red.

Yours faithfully,

Hui Yu

Reviewer #1:

The authors of the reviewed article (PCOMPBIOL-D-19-01709) aimed to catalog and characterize the mono- di- and tri-nucleotide tracts present in the reference human genome and looked for the associations of the tracts with numerous genomic features, e.g., RNA editing sites. Although, as admitted by the authors, this type of study is not completely new, however previous studies usually were focused on a particular type of repeats or particular genomic regions and often were performed in pre- or early-genomic era. Therefore, systematic complex descriptive analysis of all types (1-3 nt) of microsatellites still could have been rationalizing the study. Additionally, the authors have extended the analysis to relatively short repeat tracts (minimal length of 6, 6, and 9 nt for mono- di- and tri-nucleotide tracts, respectively) that are extremely abundant in the genome and which analysis/inclusion could have been a real novelty of the study. The study is well justified.

**Response: We are grateful for Ref. 1’s overall approval of the rationality of our study and, moreover, for the specific criticisms/suggestions raised by him/her. We took the valuable opinions of Ref. 1 into consideration and improved the work on the following major aspects: 1) we merged the complementary/redundant subgroups and thus re-defined the sub-categories of polytracts; 2) in Polytrap, we implemented an optional testing directionality for under-representation, and we also briefly presented the under-representation test results for RNA-editing events; 3) we publicized polytract data files on a stable public page; 4) we cleaned up our inconsistent terms and adopted the suggested MNR/DNR/TNR nomenclature throughout. We hope Ref. 1 will find significant improvement of this work.**
Nevertheless, in my opinion, the aspect of descriptive characterization of the repeats is not fully explored and is not informatively presented. The manuscript has also several serious drawbacks that would have to be clarified/corrected before considering the manuscript for publication.
1. The redundant repeats should be merged as it is done for triplets. There is no need to distinguish complementary tracts (e.g., A and T or GA and TC) unless located and considered in the context of the direction of a gene or other genomic elements (it is not the case of the present version of the study). Otherwise, the genomic orientation of repeat tracts is random, for example, please see almost identical statistics for complementary tracts in Appendix 1. The distinguishing the complementary tracts makes a lot of analyses redundant and unnecessarily confusing.

**Response: we have merged complementary tracts for MNRs and DNRs, as we did for TNR. Now, MNRs are divided into two groups: A/T monomers and C/G monomers; DNRs are divided into four groups: TA, CT/GA, CA/GT, and GC. Please note that the nucleotide permutation within a DNR unit does not matter, because we allow the tract to begin or end with either letter. In summary, the pruned catalog of polytracts consists of 2 types of MNRs, 4 types of DNRs, and 10 types of TNRs.**
2. The authors very inconsistently use names, descriptions for particular repeat tracts. The single/specific name should be selected for a particular tract at the beginning and then it should be used consequently throughout the entire manuscript, including pictures and supplementary materials. For example, in the Introduction the authors abbreviate trinucleotide repeats as TNRs but than use different terms, including tri-nucleotide tracts, Tri-nucleotide tracts, tri-tracts, trimers, tri, tri-, as well as again explain and use the abbreviation TNR. I would suggest to consequently to trinucleotide repeats (TNR) abbreviate mononucleotide and dinucleotide repeats (eg. MNR and DNR, respectively) and use them consequently in the manuscript.

**Response: We appreciate the practical suggestion from Ref. 1. We now adopt the MNR/DNR/TNR terminology throughout the manuscript.**
3. Results, 2nd paragraph: “We identified the same three most exome-prominent tri-tracts as previous researchers did (9): GAC, CGG, and CAG, but presenting them in a different exome-prominence order (GAC > CGG > 121 CAG, rather than CGG>CAG>GAC (9)).” In the previous study (9) the 3rd most exome prominent TNR was AGG (not GAC). GAC was the 3rd most exome overrepresented (see the difference between Fig. 1A and Fig. 1B in (9)). GAC was the least frequent TNR, with only 16 occurrences in the genome, including 3 in exons.

**Response: Thank you for carefully calibrating this point. Actually we did wish to align our result with Fig. 1B of the previous study (Kozlowski et al. 2010), so the three most exome-over-represented TNRs remained the same as GAC, CGG, and CAG. We modified our text to avoid confusion as follows.**

**“*Notably, Tri-Nucleotide Repeats (TNRs) including GAC, CGG, and CAG, as well as GC dinucleotide repeat, have substantially elevated exonic portion compared to the general pattern (Fig. 1F). The same three TNR species were found most over-represented in exome in an early survey [******1******]. Of note, GAC and CGG are the two least frequent TNR species in the genome, having chromosome density of 0.52/Mb and 12/Mb, respectively (Fig. 1C).”***
4. Results, 4th paragraph: It should be clarified whether density is a number of tracks per Mb or a number of nucleotides in tracks per Mb. It is critical for understanding the last sentence in the paragraph. To this paragraph but also generally to the study – due to the high overrepresentation of shortest tracts (extremally not normal distribution) the differences between tracks lengths are very small and therefore analyses/comparisons of means or even medians of the repeat lengths are of little value (e.g. differences in repeats lengths between chromosomes in Appendix 1.

**Response: In the first subsection of Results, we would like to make a descriptive summary of the polytract set on several common attributes, including length and density. During the summarization process, we examined if there was a significant positive correlation between the two attributes, as the ordering of polytract species seemed consistent between Fig. 1B and Fig. 1C. We did confirm a significant positive correlation between length and density for TNRs. We believe this might be a noteworthy phenomenon to present to the audience, so for result integrity we need to include the length summary. Additionally, in another subsection of Results, when we were following the early paradigm (Kozlowski et al. 2010) to investigate the length distribution patterns of various TNR species, the two TNR species of the longest mean lengths protruded, and especially AAC displayed an intriguing periodical pre-cycle length modes (Fig. 3C). We believe there are noteworthy phenomena associated with the length attribute, so we decided to keep the major points but trim the description a bit.**

5. Results, 5th paragraph, the part starting with “An intuitively plausible ...” to the end of the paragraph. I got lost in this part. Despite that I am quite familiar with the subject, it took me quite a long time to comprehend this paragraph. I would suggest to reconsider and to rewrite the paragraph.

**Response: Thank you for the feedback. We moved and rephrased that “intuitively plausible explanation” to the 3^rd^ subsection of Results, which is on enrichment of C-to-T and non-canonical RNA editing events. The rephrased narrations are as follows.**

***“In our preliminary exploration above, we noted that hinges of tandem A/T polytracts showed extremely high GC content, and now we found that C-rich polytract hinges that interrupt A/T polytracts were frequently reported to confer C-to-T RNA editing events. Taken together, they may be explained by a supposition that, in the reference genome, many cytosines (C) interrupting A/T MNRs could be thymines (T) ab initio, and the frequent C-to-T RNA editing events located at these polytract hinges were highly likely false discoveries.”***
6. Results: I do not understand the sentence ‘AAT is the most prevalent TNR species (Fig. 1E), bearing a potential replication regulatory role in partnership of 4′,6‐diamidine‐2‐phenylindole (DAPI) (15)’.

**Response: the second half of the sentence did not convey relevant information and was distracting the reader’s attention. So we removed this sentence.**
7. Results, the paragraph starting with line 196: As mentioned above, as differences in TNRs length are very small the analysis described in the paragraph is of little value. It would be more interesting and more informative to compare the frequency of TNRs occurrence in particular genomic/gene regions. It should be compared separately for each TNR type, and in genes also with consideration of genes orientation. Same for other types of repeats.

**Response: We agree with Ref. 1 that the comparison of length between different host region types is of little value. Therefore, we eliminated this analysis (the original Fig. 3C and Fig. 3D). To address the more interesting question of regional distribution of each species of polytracts, we provide pie-charts of locational distribution of each polytract species in Appendix 1: Fig. S2. In addition, in the GRCh38 polytract data file, each row is annotated as “protein-coding”, “pseudogene”, or “lncRNA”, if the polytract is located in such gene regions.**
8. Resalts, the sentence starting with line 223: Please explain the better.

**Response: Those sentences were rephrased to the following statement. And please also refer to the quoted text in response to question 5.**

***“Considering the nucleotide substitution source and target in C-to-T editing events, we may regard C/G MNRs as the ‘source’ polytracts and A/T MNRs as the ‘target’ tracts. Therefore, it is expected to see C-to-T events condense in the source, C/G MNR (Fig. 4B, ”exact tract” panel). With bi-directional 1-nt extension, however, C-to-T events demonstrate significant over-representation in both the source, C/G, and the target, A/T (Fig. 4B, “adjacency-extended tract” panel). The spurious enrichment of C-to-T events in adjacency of A/T polytracts may also be explained if we presume adjacency of A/T polytracts bears more mapping errors than the reference genome average. While the presumption of polytract hinges and adjacencies bearing more mapping errors is intuitively plausible, wet-lab experiments are needed to verify these suppositions and possibly to discriminate false C-to-T editing events.”***

9. Results, line 197: Why one-tiled test? Please justify. I think it should be the two-tiled test.
**Response: Please see our response to question 12 below.**

10. It is not always clear to me what exactly was used as a background for calculation of expected frequency or cooccurrence of repeats with particular genomic features. It is important because some repeats may associate with genomic regions enriched with the tested feature and it may lead to a spurious association. For example, it is not clarified what type of cancer somatic mutations were extracted from TCGA (whole-genome?, total whole-exome including exon flanking sequences?, only exon sequences including UTRs?, or exon ORFs excluding UTR?) and in consequence what was used as a target and background sequence for analysis. It is known that particular repeat types associates with these subregions, therefore, some comparisons may lead to false-positive spurious associations,

**Response: the expected frequency is calculated as the fraction of the collective polytract nucleotides over the total nucleotides of a genome. Polytrap does offer subtype-wise analysis, so that the collective nucleotides are quantified for a particular type of polytract or a broad group of MNR, DNR, or TNR. The newly generated schema figure (Figure 6) illustrated this idea a bit.**

**As for TCGA somatic mutations, we did additional analysis and presented the new results in following statements.**

***“Because our somatic mutation data were severely biased towards exome and certain TNR species were over-represented in exome, we were afraid the exclusive enrichment of somatic SNVs was merely a consequence of these two known biases. So we identified the subset of non-coding somatic SNVs from the total somatic SNVs and performed locational enrichment analysis on them only. Still, non-coding somatic SNVs displayed locational enrichment in TNRs only (Appendix S1: Figure S4).”***

11. Materials and Methods, ‘in human reference genomes GRCh37 and GRCh38, respectively’: What does it mean? In which assembly which repeats types have been analyzed? Why two assemblies were used?

**Response: we repeated the same identification procedure in GRCh37 and GRCh38, separately, thus obtaining cataloged polytracts in GRCh37 and GRCh38, respectively. However, we were focused on GRCh38 for the most part of the manuscript, especially in the 1^st^ subsection of Results. We identified polytracts in GRCh37 because certain genomic features we investigated, such as RNA editing events and enhancers, were located in GRCh37 instead of GRCh38.**
12. Materials and Methods, ‘H1 (alternative hypothesis) - the concerned genomic feature is distributed in polytract territory at a frequency higher than random expectation.’ Why only frequency higher than expected were considered as H1? In my opinion, both over and underrepresentations should be considered and accordingly, two-sided tests should be used. Please explain.

**Response: as we recalled in Introduction, our prior study on mitochondrial polytracts considered locational enrichment of genomic features in polytracts** [2]**, and another independent study reported characterization of dimer repeats in enhancers** [3]**. These two studies address the general question of locational enrichment a genomic feature in a defined set of genomic regions. Therefore, we made one-tailed test for the over-representation tendency the default choice of Polytrap. To accommodate occasional interest in the under-representation tendency, we added an option to Polytrap to conduct one-tailed test in the opposite tail. The relevant text modifications are as follows.**

***“Having located each polytract in the reference genome, we sought to evaluate the degree of enrichment of a particular genomic feature within the polytract territory, where the genomic feature is represented as a set of genomic sites or intervals and the polytract territory refers to the aggregate segments of the genome occupied by all polytracts (Figure 6). This is a general question of assessing the (unexpected) locational overlapping between one set of genomic intervals and another set of genomic intervals, and previous studies [******4-6******] have provided solutions of a largely same principle.”***

***“By default, Polytrap conducts a one-tailed test through the binomial distribution model, testing for the over-representation tendency of a genomic feature within the polytract territory (Eq. 1). However, we ensured Polytrap have the flexibility of testing for the under-representation tendency instead. By setting the directionality option to under-representation, Polytrap calculates the probability of reaching no more than the observed locational overlapping instances (Eq. 3), thus effectively assesses the under-representation tendency of a genomic feature within the polytract territory.”***
13. Appendix 1 and corresponding text fragments: It would be more interesting to see comparisons of particular repeat types occurrence in particular genome/gene subregions (5’UTR, 3’UTR, ORF, introns, intergenic regions) instead of in different chromosomes, in which occurrence mostly depends on chromosome length and therefore frequency or density do not differ substantially. The exception may be chromosome Y, X, and MT. Autosomes may be combined.

**Response: We converted the first tab (basic statistics of polytracts) of Appendix 1 to Table 1 in the main text, and omitted information on all other tabs. We provided comparison of genome/gene subregion representation for each of the 16 polytract species in pie-charts (Appendix: Figure S2), which partially addressed the question. Additionally, our polytract data files indicated the host gene type for each polytract, so an interested reader or user may conveniently explore or summarize the polytracts located in any of the four genome region categories: protein-coding, pseudogene, lncRNA, others.**

14. The legend in Figure 1B: What is it ‘exome splicing’? What about the coding sequence subregion? It is not indicated.

**Response: We meant a region that is in an exon or at a splicing junction (intron/exon boundaries). We changed it to “exonic/splicing” in revised Figure 1.**
15. Figure 3B and corresponding fragment of Results: Heatmap and dendrogram do not show unique distributions of CGG, ACC, and ATT. ATT show very similar distribution with AGG and quite similar distribution (same clade) with all other but CGG and ACC TNRs. The interpretation of Fig 3B should be reconsidered. Also, there is something wrong with the bitmap. It shows that the comparison of AAT with AAT (with itself) gives D-value different from 0. The same is for AGG. The same problem is with the bitmap in the supplementary Figure (Appendix 6).

**Response: We appreciate Ref. 1’s meticulous inspection of the heatmaps, which helped us fix a bug in producing the heatmaps. Now the two heatmaps show minimal color scale for all diagonal cells, consistent with the theoretical value D=0. We modified the text explanation to accord with the patterns revealed in Figs. 3A and 3B, as follows.**

***“Interestingly, this moderate-repeat-dominated dataset discriminated two TNR types inconspicuous in the prior study, namely CGG and AAC (Fig. 3, A and B). It is visually discernable that these two TNR species have the highest portions of long repeats (15U or more) than do all other species (Fig. 3A).”***
16. Figure 4A and corresponding fragments of Results. It is shown that RNA editing events are not only enriched but also underrepresented in some repeat types (eg. A2G is underrepresented in dinucleotide tracts and TNRs). The fold of these changes is similar to some positive changes but the significance of these changes is not indicated. The decreases in RNA-editing events are not commented in the text. Please explain/comment on the lack of significance of the negative changes and the fact that repeats generate both increases and decreases of RNA-editing events or artifacts.

**Response: We additionally tested for under-representation of RNA-editing events in various polytract species, and generated Figure S5 (copied below), which highlights the under-representation tendency instead.**

Figure. Locational under-representation of certain RNA-editing events in polytracts. A, barplot denotes extremity of Relative Risk (RR), with significant (p<0.01) under-representation tendency annotated with asterisk (*). B, heatmap of decreased RR of polytract bearing an RNA-editing event, depicted for combinations between editing event classes and polytract species.

**The relevant text modifications are as follows.**

***“While all analyses presented above were testing over-representation of a genomic feature, we ensured Polytrap have the flexibility of testing under-representation instead. Taking advantage of this option, we investigated the under-representation tendency of different classes of RNA-editing events in polytracts, and found A-to-G events were under-represented in almost all polytract species, excluding A/T, ACC, and hinges (Appendix S1: Figure S5).”***

***“The predominant canonical editing class, A-to-I, is under-represented in all polytract species except A/T MNR, ACC TNR, and hinges. Again, it is alerted that special caution should be cast on polytract hinge sites and boundary regions of certain repeat types.”***

17. Figure 5: It is very surprising that the significant associations ordered according to p-values correlate perfectly between repeat classes, i.e., the same order of genomic features in mono- di, and tri-nucleotide repeats. It may suggest spurious associations. I would suggest double-checking.

**Response: We think MNRs, DNRs, and TNRs displayed noticeable difference in terms of locational enrichment for miscellaneous genomic features, especially RNA editing events. Different from MNRs, TNRs show over-representation for certain genes (protein_coding genes, pseudogenes, and immunoglobulin genes), but not for any kind of RNA-editing events. DNRs are not associated with either RNA-editing events or gene regions. There are also other subtle differences, such as the enrichment of TCGA SNV in TNR only.**

18. Figure 5: It would be interesting to see also fold changes (not only p-values) for particular features. To see whether an association is positive or negative. Also, it would be interesting to see how the particular subtypes of mono-, di-, and tri-nucleotide tracts associate with the genomic features.

**Response: as we detailed in the response to question 12 above, Polytrap can now perform over-representation test and under-representation test separately. The routine output of Polytrap analysis includes a barplot of RR, from which a user will know if the association is positive or negative. Lastly, Polytrap always output subtype-wise enrichment test results, enabling the user to inspect the statistics pertaining to each of the 16 polytrap subtypes.**

19. The list of identified tracts, their type, genomic coordinates, and potentially associated genomic features should be always available for potentially interested readers. The UCSC custom tracts for the particular repeats are deposited on private google drive. I have bad experiences with “upon request” or private repositories. They usually do not work shortly after publication. Also, there is no need to mention the custom tracts for other organisms in the abstract if they are not analyzed or discussed in the paper.

**Response: We now host polytract files at a stable webpage** [**http://innovebioinfo.com/Annotation/tracts/Polytract.php**](http://innovebioinfo.com/Annotation/tracts/Polytract.php)**. Polytract files for four organisms, including human and mouse, are organized as Public Data Hubs and registered on UCSC’s Public Hubs list (**[**https://genome.ucsc.edu/cgi-bin/hgHubConnect**](https://genome.ucsc.edu/cgi-bin/hgHubConnect)**).** **We still briefly mention Polytrap’s capability regarding non-human species, because we hope this can extend Polytrap’s application to researchers working with other model organisms.**

20. Appendix 1: The title of the subtable (spreadsheet) ‘genomeOccupancy’ should be rather “chromosomeOccupancy”.

**Response: The most important message of previous Appendix 1 was converted to Table 1, and we discarded other trivial information in previous Appendix 1.**

Reviewer #2:

This paper presents an updated census of very short tandem repeats (polytracts) particularly in human reference genome GRCh38. Further, authors report their findings of enrichment and association analysis of polytracts (mainly monomers, dimers and trimers) and RNA-edit events both canonical and non-canonical with respect to various genomic features. The authors also provide polytrap, a software tool to perform such association analysis.

Overall, these findings could be of interest to a broad audience in the scientific community. However, it would be a bit of a stretch to confirm such findings when the methodology is not well presented. I would highly recommended the authors to rewrite their materials and methods to explicitly present their approach and how they conducted their analysis. In the following, I indicate a few points that should be addressed:

**Response: we appreciate the critical review of our manuscript by Ref. 2. In light of the specific advices of Ref. 2, we explained our polytract identification/categorization/analysis methods in greater details. A new figure (Figure 6) was generated to illustrate the schema of Polytrap and explain the locational enrichment test.**

• There are fundamentally major weaknesses in this manuscript:

o Authors claim that previous analysis of polytracts are outdated and imprecise. I find the argument of improved sequencing technology and better curation of the current version GRCh38 reference genome more plausible, but beside that, the authors fail to present evidence of how their analysis has led to improved detection and characterization.

**Response: we noticed that most previous works were relying on some sophisticated algorithms to detect customized sets of STRs, all of varied length and species parameters. The algorithms that were employed, such as TRF and TrStalker, allowed for imperfect or fuzzy STRs. Therefore, these approaches were fundamentally different from our Polytract set by design, and a direct comparison of the different result datasets seems unreasonable.**

**UCSC Microsatellite data track is comparable to a subset of our polytracts, and we included this comparison in the revised manuscript. We found that our dataset contains all UCSC data entries as well as qualified repeats missed by UCSC dataset. Please find more details on this comparison in our response to the first question of Ref. 3 (below). Additionally, we compared the polytract sets generated between GRCh38 and GRCh37, both resulting from the same detection method and parameter set. We found that a greater volume of polytracts were observed in GRCh38. The two new comparisons were described in Results as follows.**

***“Simply comparing the numbers, this latest percentage of 4.7% is much larger than previous estimations of ~3% that were based on early reference genomes [******7******,*** ***8******]. Because the minimum length of STRs was not set identically and the encompassed STR species were not the same, these percentages were not directly comparable to each other. However, we were able to compare these statistics between GRCh38 and GRCh37 in parallel. Using the same protocol, we identified all polytracts in the reference genome GRCh37. Surprisingly, while the total genome size reduced by 0.2% from GRCh37 to GRCh38 [******9******], the nucleotide volume occupied by MNRs, DNRs, and TNRs C increased in absolute number (Appendix S1: Table S1); in total, GRCh37 and GRCh38 harbor 141.6 million (4.6%) and 144.4 million (4.7%) polytract nucleotides, respectively. It seemed that broader regions of polytracts are revealed in newer, more refined reference genomes.”***

***“In the field, there is a similar data resource of GRCh38 DNRs/TNRs available as UCSC Microsatellites (***[***http://genome.ucsc.edu/cgi-bin/hgTrackUi?hgsid=104818371&c=chrX&g=microsat***](http://genome.ucsc.edu/cgi-bin/hgTrackUi?hgsid=104818371&c=chrX&g=microsat)***). We extracted a comparable subset of DNRs and TNRs from our polytract dataset and compared it with the UCSC Microsatellite dataset. We found that literally 100% of UCSC Microsatellites were recovered in the polytract set, but the latter contained additional qualified repeat instances missed by the former (Appendix S1: Table S2).”***

o Authors do not provide the theoretical framework behind the identification, detection and categorization of polytracts. Authors relied on BSgenome, a Bioconductor package. I find the details in Methods and Materials insufficient in terms of polytracts detection and identification. Authors must include the underlying logical framework and methodology used to infer polytracts from a DNA sequence (reference genome). Authors should indicate whether the method considered leads to improved detection of polytracts especially monomers and dimers that have not been widely reported.

**Response: Unlike the widely-used repeat finder TRF** [10] **or several of its modified versions, we did not attempt to identify fuzzy or imperfect repeat tracks. We extracted only perfect tandem matches to the specified motif. That means a tract in the form of “CAGCAGCAGCAG” meets our standard while a slightly disrupted tract “CAGCTGCAGCAG” does not. The latter, however, may well qualify as a tandem repeat in the perspective of TRF or TrStalker, which allow a degree of divergence/interruption of the unit motif. So, identification of our defined polytracts is pretty straightforward – it is basically a string match of a pattern against each full-length chromosome sequence. We did a comparison of our DNRs (Dinucleotide repeats) and TNRs against the comparable ones from UCSC Microsatellites, and found that our dataset contains all UCSC data entries as well as qualified repeats missed by UCSC dataset. Please find more details on this comparison in our response to the first question of Ref. 3 (below).**

**The relevant textual modification is as follows.**

***“A polytract is defined as a tract of mono-nucleotide, di-nucleotide, or tri-nucleotide tandemly repeated motifs, with possibly incomplete terminal motif included, where the minimum number of repeated units are 6 for MNR and 3 for DNR and TNR. Unlike many related works which allow fuzzy STRs, we seek only perfect matches to our definition. This was done through a string matching between a pattern and each chromosome sequence, with assistance from R packages “stringr” (***[***https://CRAN.R-project.org/package=stringr***](https://CRAN.R-project.org/package=stringr)***) and “BSgenome.Hsapiens.UCSC.hg38” (***[***www.bioconductor.org***](http://www.bioconductor.org)***; DOI:*** [***10.18129/B9.bioc.BSgenome***](https://doi.org/doi:10.18129/B9.bioc.BSgenome)***).”***

o The paper as it is now has no details or description of the statistical analysis for the results presented. Authors should include a section describing and discussing the statistical analysis and statistical tests performed.

**Response: we augmented and refined the subsection of Materials and Methods, “Genomic features enrolled in the locational enrichment analysis.” A whole new paragraph was inserted at the beginning of this section. Visual illustration of the enrichment test is provided in the new Figure 6.**

o Authors present polytrap and yet have not described its algorithmic idea and how it works. I encourage the authors to include such information as well.

**Response: We created a schema figure (Figure 6) to explain the algorithmic idea of Polytrap, and expanded the original descriptions to one individual subsection of Results, titled “A software package to evaluate genomic feature enrichment within polytracts.”**

• The Authors use the binomial distribution and clearly express the hypotheses they wish to test. The authors should explain why they considered this distribution and any underlying assumptions made and how this relates to the downstream association analysis.

**Response: we added one paragraph to describe the problem more clearly and explain our choice of binomial distribution. This new paragraph is as follows.**

***“Having located each polytract in the reference genome, we sought to evaluate the degree of enrichment of a particular genomic feature within the polytract territory, where the genomic feature is represented as a set of genomic sites or intervals and the polytract territory refers to the aggregate segments of the genome occupied by all polytracts (Figure 6). This is a general question of assessing the (unexpected) locational overlapping between one set of genomic intervals and another set of genomic intervals, and previous studies [******4-6******] have provided solutions of a largely same principle. Basically, we define the expected frequency of observing the genomic feature within the polytract territory as n/N, where N denotes the total size of the genome and n the size of the polytract territory; when k out of M instances of the genomic feature fall into the polytract territory, if the observed frequency k/M is much higher than n/N it indicates probable enrichment. Classical probability models such as hypergenometric model and binomial model are frequently used in such scenarios to test for locational enrichment. Here, we followed early examples [******4******,*** ***6******] to employ the binomial model.”***

o How do you define and determine the tract territory?

**Response: The polytract territory means the aggregate segments of the genome occupied by all polytracts. When we focus on the whole set of polytracts, the polytract territory refers to the 144.4 million nucleotides occupied by all MNRs, all DNRs, and all TNRs. When we focus on just one group of polytracts, say TNRs, the polytract territory refers to the 14.7 million nucleotides occupied by all TNRs.**
o L387-L392: The singleton mode and the multiplex mode, it should be helpful to visually illustrate the different modes if possible.

**Response: We now depict the two different modes through visual illustration in Figure 6 (bottom left part).**
o Authors use terms like tract number, density, tract volume and genome occupancy and yet do not explain them in the main text nor in the supplementary materials [Supp. S1].

**Response: We proofread the revised manuscript thoroughly to ensure use of consistent, defined terms. Below are example introductions/definitions of some terms.**

***“Even more evident disparity is found with tract density, defined as number of polytracts per Mb.”***

***“In a nutshell, we evaluated if RNA editing events were over-represented in the polytract territory as compared to the baseline frequency across the whole genome. Here, the polytract territory referred to the aggregate segments of the genome occupied by all polytracts, and it was their collective nucleotide volume that we quantified.”***

• Location of miscellaneous genomic features
o Authors should briefly discuss why they considered the specified genomic features and discuss briefly their biological importance for instance with respect to RDDs

***Response: We added these sentences to explain our reasons of selecting those genomic features –***

***“Ninety-four genomic features located in GRCh38 or GRCh37 were coarsely grouped to five major classes, namely RNA-DNA difference (RDD), enhancer, Gene region in HG38, RNA-binding protein’s binding segments, and miscellaneous. We recruited RDD foremost because RDDs displayed an intriguing enrichment in mitochondrial polytracts [******2******] and we wanted to verify if the same phenomenon exists in the nuclear genome. Enhancer was chosen because an early study discovered characterization of drosophila enhancer with DNR [******3******] and we wanted to verify if the same phenomenon exists in the human genome. Diverse gene regions in HG38 were separately analyzed to yield a comparative view of polytract distribution across different types of gene regions. Certain RNA-binding proteins’ binding motifs manifest inherent nucleotide repeats, so the imminent analysis would be able to confirm such internal repeat feature and also possibly indicate nearby or distant repeat features. Finally, more than ten miscellaneous features were included the analysis because they were sequential features with easily attainable genomic locations.”***
o It appears to me that the authors only listed materials gathered for their analysis. They fail to present how they actually conducted and performed their analysis. You have collected data from various sources with potentially different genomic coordinates (GRCh37 vs GRCh38, for instance) and yet you do not mention how all that information was integrated and used.

**Response: We did identify polytracts in the same fashion in GRCh38 and GRCh37, respectively. We did not attempt to integrate the result sets across distinct reference genomes. As we indicated in subsection “Genomic features enrolled in the locational enrichment analysis,” data sources for the myriad of genomic features may be based on either GRCh38 or GRCh37, and we just performed locational enrichment analysis of the genomic feature with respect to the genome version it was localized in. For instance, enhancers and RNA editing sites were located in GRCh37, and we performed their enrichment analysis using the GRCh37 polytracts; target 3’-UTR segments of RNA binding proteins were located in GRCh38, and we performed their enrichment analysis using the GRCh38 polytracts. We wrote these sentences to clarify this treatment –**

***“In the same manner as we dealt with the reference genome GRCh38, we identified all polytracts in the reference genome GRCh37.” … “In the following analyses, certain data resources (including enhancers and RNA editing sites) were based on GRCh37 instead of GRCh38, and in such cases we performed the analyses against GRCh37 polytracts.”***

o Overall, the materials and method part of the paper needed to be significantly improved

**Response: we addressed the specific points concerning the Materials and Methods part and augmented it significantly. A new figure (Figure 6) was added to illustrate the schema of Polytrap and the principle of enrichment test.**

• Minor issues:
o Provide citation L151

**Response: It was done.**

o L215: For consistency, authors should report their RR as done at L223 also to avoid confusion with citation notations.

**Response: It was done.**

o L323-L329: I would encourage authors to discuss and put forth the argument of why analysis of A2G editing events are needed in the context of host polytract and probably mention their current manuscript in preparation rather than reporting their findings.

**Response: we thought the preliminary data needs further investigation and was unsuitable to appear in the Discussion part. So we removed those statements.**

o Proofread the text
**Response: It was done.**

o The figures could be a of a better quality and higher resolution

**Response: It was done.**

Reviewer #3:

This article is well written and thoroughly tries to review/census monomers, dimers, and trinucleotide (tandem) repeats found in GRCh38, by correlating their distribution and composition to specific genomic features and loci.

**Response: we appreciate the critical review of our manuscript by Ref. 3. According to Ref. 3’s specific comments, we tackled the inconsistency problem in terms of reverse complement and in consequence re-defined the sub-categories of polytracts. The resultant polytract data files, and all figures/tables were modified accordingly to concord with the adjusted categorization. Also, we conducted a comparison between our polytract dataset and UCSC Microsatellites and confirmed a better quality of our data. The Discussion was substantially improved by incorporating the insights from the referee.**

However, as a first observation, no comparison is made with, for example, simple repeats (UCSC track, last updated on March 2019) and works associating them with human diseases and other genomic features, as well as with the mentioned 700k catalog by Willems et al. and other catalogues and censuses that can be easily retrieved from online (e.g. <https://www.frontiersin.org/articles/10.3389/fgene.2018.00155/full>).

**Response:**

**UCSC Simple Repeats are comprised of perfect or imperfect tandem repeats, which were outputted by a well-known software TRF (**[**http://tandem.bu.edu/trf/trf.submit.options.html**](http://tandem.bu.edu/trf/trf.submit.options.html)**). From all UCSC Simple Repeats, “sequences of at least 15 perfect di-nucleotide and tri-nucleotide repeats” were extracted to form a separate track of Microsatellites. By definition, UCSC Microsatellites are comprised of perfect TNRs and DNRs, which are theoretically comparable to our polytracts. Therefore, we extracted subsets of TNRs and DNRs of 15+ unit length from our polytract set, and examined bilateral overlapping between UCSC tracks and our data. The “overlapping” between interval A and interval B is approved when they are located on a same chromosome and they share at least one nucleotide. As shown in Table 1, literally 100% of UCSC microsatellites are recovered in the Polytract set, but the latter always contain additional repeat instances. Overall concordance between the two sources is reflected by highly similar trend of density running along chromosomes (Figure below. A). We manually inspected several Polytract-specific TNRs and did confirm their qualification. For example, a repeat of 15 tandem copies of “GAG” on chr1:106,731,004~106,731,050 is captured in the Polytract set only, missing in the UCSC Microsatellite set (Figure below. B).**

**The 700k catalog by Willems et al. and a most recent repeat census by Genovese et al. were generated through TRF inference followed by tedious post-processing. Like UCSC Simple Repeats, they are fundamentally different from our Polytract set by design, so we did not perform a comparison against them. But we modified our Introduction to explain the different logics, as follows.**

**“*In the past decade, although many efforts were dedicated to calling STR variations from Next-Generation Sequencing data, an updated census of STR in the human reference genome is surprisingly unobserved. As a preparation step to their major analyses, two studies [******11******,*** ***12******] compiled STR catalogs in GRCh37 and GRCh38, respectively; both works employed ad hoc procedures and custom parameters, thus generating highly customized result sets unsuitable for general use. In particular, both catalogs were initially generated by an inference-based algorithm TRF [******10******], so by definition they represented a mixture of perfect and imperfect STRs*.”**

**Table. Numbers of repeats in the UCSC microsatellite set, the Polytract set, and their overlapping set.**

| **Repeat category** | **Polytract** | **Microsatellite** | **Polytract As Microsatellite** | **Polytract As Microsatellite (%)** | **Microsatellite As Polytract** | **Microsatellite As Polytract (%)** |
| --- | --- | --- | --- | --- | --- | --- |
| aag | 1514 | 55 | 159 | 11% | 55 | 100% |
| agg | 121 | 13 | 36 | 30% | 13 | 100% |
| acc | 131 | 19 | 52 | 40% | 19 | 100% |
| ct | 2575 | 1337 | 1337 | 52% | 1337 | 100% |
| ga | 2591 | 1384 | 1384 | 53% | 1384 | 100% |
| ta | 11992 | 6891 | 6891 | 57% | 6891 | 100% |
| cgg | 33 | 7 | 19 | 58% | 7 | 100% |
| cag | 86 | 19 | 56 | 65% | 19 | 100% |
| atc | 155 | 46 | 101 | 65% | 46 | 100% |
| act | 89 | 24 | 65 | 73% | 24 | 100% |
| ca | 20646 | 15279 | 15279 | 74% | 15279 | 100% |
| gt | 20853 | 15538 | 15538 | 75% | 15538 | 100% |
| aac | 217 | 74 | 183 | 84% | 74 | 100% |
| aat | 1899 | 654 | 1618 | 85% | 654 | 100% |

Figure. Comparison between UCSC Microsatellites and Polytracts. A, general concordance between UCSC Microsatellites and Polytracts is reflected by similar running density along chromosomes. B, a specific TNR instance of 15 copies of “GAG” is captured in the Polytract set but not the UCSC Microsatellite set.

More importantly, no accurate details and definitions are given for the discovery of such (tandem) repeats, while these could clarify some of the statistical findings and help identifying potential biases with the analysis; sophisticated software and methodologies are nevertheless available from other works, which allow for a flexible and robust tandem repeat finding (e.g. TRF and more recent tools). Reverse complement is also only considered for trinucleotides, while the analysis should be comparable between all three types for some statistics.

Response:

**We modified the Introduction and Methods to describe the fundamental difference between our identification method and common existent approaches. We also merged complementary tracts for MNRs and DNRs, as we did for TNR. Now, MNRs are divided into two groups: A/T monomers and C/G monomers, and DNRs are divided into four groups: TA, CT/GA, CA/GT, and GC. Please note that the permutation order within a DNR does not matter, because we allow the tract to begin or end with either letter. In summary, the pruned catalog of polytracts consists of 2 types of MNRs, 4 types of DNRs, and 10 types of TNRs.**

**The relevant textual modifications are as follows.**

***“Different from related works, our polytract catalog is intentionally biased towards very short STRs which comprise MNRs, DNRs, and TNRs, and it includes only perfect matches to our polytract definition (see Materials and Methods).”***

***“A polytract is defined as a tract of at least six tandem repeats of mono-nucleotide, di-nucleotide, or tri-nucleotide motif, with possibly incomplete terminal motif included. Unlike many related works which allow fuzzy STRs, we seek only perfect matches to our definition. This was done through a string matching between a pattern and each chromosome sequence, with assistance from R packages “stringr” (***[***https://CRAN.R-project.org/package=stringr***](https://CRAN.R-project.org/package=stringr)***) and “BSgenome.Hsapiens.UCSC.hg38.” Because of the complementarity between a purine and pyrimidine pair (A:T and G:C), we combined poly-A and poly-T runs into an “A/T” group, poly-G and poly-C runs into a “G/C” group, poly-CT and poly-GA runs into a “CT/GA” group, and poly-CA and poly-GT runs into a “CA/GT” group. Similarly, a nominal whole set of 60 TNR motifs were merged into 10 groups : AAC (accommodating AAC, ACA, CAA, GTT, TGT, and TTG), AAG (AAG, AGA, GAA, CTT, TCT, and TTC), AAT (AAT, ATA, TAA, ATT, TAT, TTA), ACC (ACC, CCA, CAC, GGT, TGG, GTG), GAC (GAC, ACG, CGA, GTC, CGT, and TCG), ACT (ACT, CTA, TAC, AGT, TAG, and GTA), CAG (CAG, AGC, GCA, CTG, GCT, and TGC), AGG (AGG, GGA, GAG, CCT, TCC, and CTC), ATC (ATC, TCA, CAT, GAT, TGA, and ATG), and CGG (CGG, GGC, GCG, CCG, GCC, and CGC). In summary, our resultant polytract dataset consisted of two types of MNRs, four types of DNRs, and ten types of TNRs (Table 1). ”***

Furthermore, the fact that the main differences between DNA and RNA are found inside or adjacent to monomer repeats might easily suggest that these come either from sequencing errors, alignment consensus errors, or possible (low-frequency) polymorphisms. Indeed, monomers, especially long monomers, can be hardly sequenced well and are known to create several artefacts and analysis issues both in sequencing, pre-processing, and post-processing phases, as well as are more prone to indels of a different nature (than the repeat itself) than the other two types. Not surprisingly, significant differences in the distribution of somatic mutations in cancer genomes is noted for both them and trinucleotides (i.e. the size of codons). I would thus suggest to avoid, at least for the moment, mainly focusing on these discoveries both in the title and the abstract.

**Response: We incorporated Ref. 3’s insights here into our Discussion, as pasted below.**

***“In accordance with its inherent error-prone nature, polytracts demonstrate enrichment of non-canonical RNA-editing events, somatic indels, and blacklist variants. Thus, polytracts can reasonably serve as a negative reference set for quality controlling genome/exome sequencing studies, helping to flag suspicious variant calls or other sequencing results that are significantly biased to polytract regions. For this negative quality control purpose, we suggest more weights be given to MNR than DNR and TNR, because monomer runs are especially intractable regions in sequencing experiments thus a lower data quality may be associated with MNR in terms of both the mapped sequence in the reference genome and the re-mapped sequence in a personal genome. Across the comparative enrichment landscapes of MNR/DNR/TNR, somatic SNVs are enriched in TNR only whereas somatic indels are enriched in all three polytract clades. In practical sequencing projects, downstream analyses in succession to variant calling usually trust SNVs more than indels, and a component of too many indels raises a warning sign. The disparate polytract enrichment pattern between SNV and indel is consistent with our varied confidence towards SNV and indel in sequencing practices. Likewise, the blacklist variants display the highest statistical significance of enrichment in MNR yet the lowest statistical significance in TNR, again discriminating the more error-prone nature of MNR. So, it is advisable that sequencing practitioners include polytract/MNR enrichment analysis in their quality control protocols.***

***Polytracts comprise DNRs and TNRs in addition to MNRs. TNRs are appreciated more as biologically significant motifs than avoidable pitfalls. In the polytract enrichment landscapes, TNR does show significant enrichment for many meaningful cis-elements, including transcription-factor binding sites and RNA-binding protein target segments. Interestingly, many genomic features are enriched in MNR/DNR as well as in TNR. Especially, DNR resembles TNR in demonstrating strong over-representation of enhancers and strong under-representation of A-to-I RNA-editing events, thus indicative of remarkable biological significance. MNR also shows significant enrichment for some meaningful genomic features, including binding targets for select RNA-binding proteins. RNA-binding proteins HNRNPC, TIA1, and U2AF2 all favor U-rich binding motifs and their target sequences show distinctively lower entropy than common RNA-binding proteins [******13******]; concordantly, binding segments of these three RNA-binding proteins enrich MNR yet not DNR or TNR (Fig. 6). In this context, the enrichment within MNR indicates differential characteristics of binding motifs of select RNA-binding proteins. Therefore, it risks neglecting genuine biological signals if we always ignore or downweight features/entities that are enriched in simple repeats such as MNRs. Generally speaking, domain knowledge and meticulous inspection are necessary for interpreting polytract enrichment results rationally. ”***

Finally, no information is available on the created software, as well as on its memory footprint and general performance.

**Response: we created a schema figure (Figure 6) to explain the algorithmic idea of Polytrap, and expanded the original descriptions to one individual subsection of Results, titled “A software package to evaluate genomic feature enrichment within polytracts.” Lastly, we recorded the time and memory usage pertinent to RNA-editing analyses (Table 3) to enable estimation of practical computational costs, which is proportional to the size of the custom dataset.**

Reviewer #4:

Based on the new human reference genome GRCh38, this manuscript studied the genome-wide short tandem repeats (STR) characteristics, in particular the monomer, dimer, and TNR and the implication on RNA-DNA differences and other genome features, which has provided new insight into our understanding of the impact of STR on genome structure and function. In addition, they developed a software to characterize STRs in the genomes, which will be helpful for other researchers. However, some questions are not reasonable and needed to be clarified.

**Response: we appreciate the critical review of our manuscript by Ref. 4. Per the suggestions, we clarified our polytract detection methods and explained the different motivations between our work and related works. A brief comparison between GRCh38 and GRCh37 was added. Discussion was thoroughly re-wrote to highlight the major findings.**

Major comments:

1.The authors used infrastructure packages from Bioconductor ([www.bioconductor.org](http://www.bioconductor.org);
DOI: 10.18129/B9.bioc.BSgenome) to identify monomer, dimer, and TNR in human reference genomes，and found the abundance of very short tandem repeats in the new reference genome was much higher than those in the previous reference genome. I am wondering why the authors did not apply some traditional methods such as RepeatSeq or lobSTR to recognize STRs to the reference genome? The authors should justify their methodology and figure out whether the new version of reference genome or the data procession cause such differences on STR abundance. The authors also can check this literature to find better way to deal with STRs.
Willems T, Zielinski D, Yuan J, Gordon A, Gymrek M, Erlich. Genome-wide profiling of heritable and de novo STR variations. Nature Methods. 2017, 14, 590–592.

**Response: The few methods recommended by Ref. 4, including RepeatSeq, lobSTR, and HipSTR, are purported to call repeat variance reliably from Next Generation Sequencing data. The first step of our work is to identify the repeat tracks existent within the reference genome GRCh38. The two tasks are different, so we did not employ any of these methods. We modified our Introduction to clarify our foremost step of polytract identification, as follows.**

***“In the past decade, although many efforts were dedicated to calling STR variations from Next-Generation Sequencing data, an updated census of STR in the human reference genome is surprisingly unobserved.”***

***“We felt a pressing need to expand the analysis of tract-associated genomic features to the whole contemporary human reference genome (GRCh38), aligning the under-investigated MNRs and DNRs with the more established TNRs. For this purpose, we first created an up-to-date catalog of precisely defined very short tandem repeats, including MNRs, DNRs, and TNRs, which are termed “polytracts” in general.”***

***“A polytract is defined as a tract of at least six tandem repeats of mono-nucleotide, di-nucleotide, or tri-nucleotide motif, with possibly incomplete terminal motif included. Unlike many related works which allow fuzzy STRs, we seek only perfect matches to our definition. This was done through a string matching between a pattern and each chromosome sequence, with assistance from R packages “stringr” (***[***https://CRAN.R-project.org/package=stringr***](https://CRAN.R-project.org/package=stringr)***) and “BSgenome.Hsapiens.UCSC.hg38” (***[***www.bioconductor.org***](http://www.bioconductor.org)***; DOI:*** [***10.18129/B9.bioc.BSgenome***](https://doi.org/doi:10.18129/B9.bioc.BSgenome)***).”***

***“STRs are receiving increasing appreciation for their roles as genetic markers, polymorphism variants, origins of heritable neurological diseases [******14******]. Recently, several analysis tools were developed to facilitate STR variant calling from sequencing data [******15-17******]. Among the increasing research efforts, we noted a lack of systematic census and characterization of STRs in the updated human reference genome. A careful scrutiny and characterization of STRs in the reference genome will benefit the expanding STR-focused personal genome analyses.”***

2.Considering a large number of previous studies on human genome STRs and their characteristics, it is suggested that the author supplement the differences between GRCH38 and previous version, and add the comparison of their results with other studies based on the old version of reference genome.

**Response: the polytract identification process was repeated between CRCh38 and GRCh37 in parallel, and both sets of polytracts are included in the publiczed datasets. We generated Table S1 to exhibit comparative summary statistics of polytracts between GRCh38 and GRCh37, and added the following statements in Results to touch on the difference between these two reference genome versions. However, it would entail too much work if we systematically expanded all analyses from GRCh38 to GRCh37.**

***“Simply comparing the numbers, this latest percentage of 4.7% is much larger than previous estimations of ~3% that were based on early reference genomes [******7******,*** ***8******]. Because the minimum length of STRs was not set identically and the encompassed STR species were not the same, these percentages were not directly comparable to each other. However, we were able to compare these statistics between GRCh38 and GRCh37 in parallel. Using the same protocol, we identified all polytracts in the reference genome GRCh37. Surprisingly, while the total genome size reduced by 0.2% from GRCh37 to GRCh38 [******9******], the nucleotide volume occupied by MNRs, DNRs, and TNRs unexclusively increased in absolute number (Appendix S1: Table S1); in total, GRCh37 and GRCh38 harbor 141.6 million (4.6%) and 144.4 million (4.7%) polytract nucleotides, respectively. It seemed that broader regions of polytracts are revealed in newer, more refined reference genomes.”***

3.Although the detail analysis has been conducted on the GRCH38 reference genome in the present study, it is only based on one genome. As known for us, short tandem repeat sequences show high polymorphism between genome and genome. How can an analysis based on a single genome reflect the real characteristics of STRs in human or how high is the reliability? The authors should clarify.

**Response: As we clarified in the beginning of Discussion, *“STRs are receiving increasing appreciation for their roles as genetic markers, polymorphism variants, origins of heritable neurological diseases [******14******]. Recently, several analysis tools were developed to facilitate STR variant calling from sequencing data [******15-17******]. Among the increasing research efforts, we noted a lack of systematic census and characterization of STRs in the updated human reference genome. A careful scrutiny and characterization of STRs in the reference genome will benefit the expanding STR-focused personal genome analyses.”***

4.Based on the new reference genome, the authors analyzed the RNA editing sites associated with STRs, which is one of the highlights of this manuscript. It is suggested that the authors should strengthen this in the Results, in particular they should add some comments in the Discussion to highlight the significance of STRs on RNA editing events in combination with relevant literatures on human RNA editing in recent years.

**Response: a single paragraph is dedicated to discussing the significance of RNA-editing-related observations in the present work, as pasted below.**

***“RNA-editing events are classified as canonical events and non-canonical events, and A-to-I events dominate over C-to-U events in the canonical category. There has been a long-lasting dispute over the authenticity of non-canonical editing events [******18******]. Peer researchers pointed out flaws in designs or protocols of studies that advocate the universality of non-canonical editing events [******19******,*** ***20******]. Here, we discovered that non-canonical RNA-DNA differences are overrepresented in MNR adjacent sites or hinge sites whereas A-to-I events are generally under-represented in polytracts. From a novel angle, the selective enrichment of non-canonical RNA-editing events within MNR adjacency provides a negative evidence against their authenticity, and it also supports the practical operation of assessing the quality of an RDD dataset by the proportion of A-to-I events [******21******]. We even argue that a considerable portion of C-to-T editing events are false discoveries, as C-to-T editing events are condensed in the vicinity and hinge sites of polytracts, resembling non-canonical events but not A-to-I events. Our suspicion on C-to-T editing events might be linked to a recent finding that human C-to-U coding RNA editing is largely nonadaptive and that they probably manifest cellular errors [******22******]. The predominant canonical editing class, A-to-I, is under-represented in all polytract species except A/T MNR, ACC TNR, and hinges. Again, it is alerted that special caution should be cast on polytract hinge sites and boundary regions of certain repeat types.”***

5.The manuscript developed a software to assist with custom association analysis of polytracts with respect to any localizable genomic feature. Unfortunately, trying to assist the Polytrap is failed to me. Furthermore, the link provided by the manuscript about genomic coordinates of polytracts in nine model organisms is also unavailable. Please provide validated way for using and evaluating the Polytrap. And i would like to know if the Polytrap could be used for population scale analysis.
**Response: we apologize for the inaccessibility of Polytrap. Polytrap is hosted in GitHub and should be generally accessible to academic professionals. We had internal and external collaborators test the download, installation, and trial sessions. It is in good shape now.**

**The input to Polytrap can be typical analysis results of sequencing projects, such as SNVs, indels, CNVs, etc. Alternatively, it can be established genomic datasets represented technically as sets of genomic segmental intervals (start and end coordinates on chromosomes), like the datasets for Retrotransposons Insertion Polymorphisms or RNA-editing events that we investigated in the present study. If a population study entails such a dataset, Polytrap is applicable.**

6.There are many recent literatures about STRs or RNA editing in human genome. In the discussion part, the authors seldom used the literatures and compared their results with the previous studies. Many sentences in the discussion are repeat the results, thus suggest the authors to improve.

**Response: We thoroughly re-wrote the Discussion part, replacing the redundant statements with novel discussions. Additional citations, especially No. 24-30, were included to strengthen these discussions.**

Minor comments:
In the Abstract and Keywords, the authors used C-to-U RNA-editing, however in the text part, they used A2T or C2T. This should be revised.

**Response: We have unified these to A-to-G and C-to-T throughout.**

**Have all data underlying the figures and results presented in the manuscript been provided?**
Large-scale datasets should be made available via a public repository as described in the *PLOS Computational Biology* [data availability policy](http://journals.plos.org/ploscompbiol/s/data-availability), and numerical data that underlies graphs or summary statistics should be provided in spreadsheet form as supporting information.

Reviewer #1: No: The lists of identified mono-, di-, and tri-nucleotide tracts provided as custom track files for UCSC Genome Browser are deposited (linked) only on private Google Drive. These lists are very important result of the study and should be deposited in public database or if possible as supplementary materials.

Reviewer #2: Yes

Reviewer #3: Yes

Reviewer #4: No: the polytrap is unaccessable

PLOS authors have the option to publish the peer review history of their article ([what does this mean?](https://journals.plos.org/ploscompbiol/s/editorial-and-peer-review-process#loc-peer-review-history)). If published, this will include your full peer review and any attached files.


If you choose “no”, your identity will remain anonymous but your review may still be made public.


**Do you want your identity to be public for this peer review?** For information about this choice, including consent withdrawal, please see our [Privacy Policy](https://www.plos.org/privacy-policy).

Reviewer #1: No

Reviewer #2: No

Reviewer #3: No

Reviewer #4: No

*In compliance with data protection regulations, you may request that we remove your personal registration details at any time.* [*(Remove my information/details)*](https://www.editorialmanager.com/pcompbiol/login.asp?a=r)*. Please contact the publication office if you have any questions.*

1.	Kozlowski P, de Mezer M, Krzyzosiak WJ: **Trinucleotide repeats in human genome and exome**. *Nucleic acids research* 2010, **38**(12):4027-4039.

2.	Zhao S, Samuels DC, Zhao YY, Guo Y: **Tri-allelic heteroplasmies, DNA-RNA differences and their polynucleotide tract associations in the mitochondrial genome**. *Genomics* 2017.

3.	Yanez-Cuna JO, Arnold CD, Stampfel G, Boryn LM, Gerlach D, Rath M, Stark A: **Dissection of thousands of cell type-specific enhancers identifies dinucleotide repeat motifs as general enhancer features**. *Genome research* 2014, **24**(7):1147-1156.

4.	McLean CY, Bristor D, Hiller M, Clarke SL, Schaar BT, Lowe CB, Wenger AM, Bejerano G: **GREAT improves functional interpretation of cis-regulatory regions**. *Nature biotechnology* 2010, **28**(5):495-501.

5.	Otlu B, Firtina C, Keles S, Tastan O: **GLANET: genomic loci annotation and enrichment tool**. *Bioinformatics* 2017, **33**(18):2818-2828.

6.	Ward LD, Kellis M: **HaploReg: a resource for exploring chromatin states, conservation, and regulatory motif alterations within sets of genetically linked variants**. *Nucleic acids research* 2012, **40**(Database issue):D930-934.

7.	Subramanian S, Mishra RK, Singh L: **Genome-wide analysis of microsatellite repeats in humans: their abundance and density in specific genomic regions**. *Genome biology* 2003, **4**(2):R13.

8.	Gymrek M: **A genomic view of short tandem repeats**. *Current opinion in genetics & development* 2017, **44**:9-16.

9.	Guo Y, Dai Y, Yu H, Zhao S, Samuels DC, Shyr Y: **Improvements and impacts of GRCh38 human reference on high throughput sequencing data analysis**. *Genomics* 2017, **109**(2):83-90.

10.	Benson G: **Tandem repeats finder: a program to analyze DNA sequences**. *Nucleic acids research* 1999, **27**(2):573-580.

11.	Willems T, Gymrek M, Highnam G, Genomes Project C, Mittelman D, Erlich Y: **The landscape of human STR variation**. *Genome research* 2014, **24**(11):1894-1904.

12.	Genovese LM, Geraci F, Corrado L, Mangano E, D'Aurizio R, Bordoni R, Severgnini M, Manzini G, De Bellis G, D'Alfonso S *et al*: **A Census of Tandemly Repeated Polymorphic Loci in Genic Regions Through the Comparative Integration of Human Genome Assemblies**. *Frontiers in genetics* 2018, **9**:155.

13.	Sheng Q, Yu H, Oyebamiji O, Wang J, Chen D, Ness S, Zhao YY, Guo Y: **AnnoGen: Annotating Genomewide Pragmatic Features**. *Bioinformatics* 2020.

14.	Pai TW, Chen CM: **SSRs as genetic markers in the human genome and their observable relationship to hereditary diseases**. *Biomarkers in medicine* 2016, **10**(6):563-566.

15.	Willems T, Zielinski D, Yuan J, Gordon A, Gymrek M, Erlich Y: **Genome-wide profiling of heritable and de novo STR variations**. *Nature methods* 2017, **14**(6):590-592.

16.	Gymrek M, Golan D, Rosset S, Erlich Y: **lobSTR: A short tandem repeat profiler for personal genomes**. *Genome research* 2012, **22**(6):1154-1162.

17.	Highnam G, Franck C, Martin A, Stephens C, Puthige A, Mittelman D: **Accurate human microsatellite genotypes from high-throughput resequencing data using informed error profiles**. *Nucleic acids research* 2013, **41**(1):e32.

18.	Guo Y, Yu H, Samuels DC, Yue W, Ness S, Zhao YY: **Single-nucleotide variants in human RNA: RNA editing and beyond**. *Briefings in functional genomics* 2018.

19.	Lin W, Piskol R, Tan MH, Li JB: **Comment on "Widespread RNA and DNA Sequence Differences in the Human Transcriptome"**. *Science* 2012, **335**(6074).

20.	Schrider DR, Gout JF, Hahn MW: **Very Few RNA and DNA Sequence Differences in the Human Transcriptome**. *Plos One* 2011, **6**(10).

21.	Lee JH, Ang JK, Xiao X: **Analysis and design of RNA sequencing experiments for identifying RNA editing and other single-nucleotide variants**. *Rna* 2013, **19**(6):725-732.

22.	Liu Z, Zhang J: **Human C-to-U Coding RNA Editing Is Largely Nonadaptive**. *Molecular biology and evolution* 2018, **35**(4):963-969.
