# Supplementary material for: Non-canonical RNA-DNA differences and other human genomic features are enriched within very short tandem repeats
Source: PLoS Comput Biol. 2020 Jun 8;16(6):e1007968. doi: 10.1371/journal.pcbi.1007968 (PMC7302867; doi:10.1371/journal.pcbi.1007968)
Supplement: Attachment — Submitted filename: Polytract.p2pResponse.R2.docx [file pcbi.1007968.s011.docx]

April 23th, 2020

Dear Drs. Iakoucheva and Noble,

Thank you very much for asking us to revise the manuscript (PCOMPBIOL-D-19-01709R1) a second time. We carefully reviewed the remaining questions of reviewers and improved the work further based on their thoughtful suggestions. Overall, we clarified the details and corrected a minor mistake caught by Ref. 1, augmented the methodology description as requested by Ref. 2, and followed Ref. 3’s advice to ensure the additional polytracts relative to UCSC Microsatellites were correct. One additional supporting file was supplied as Appendix S2. We hope you and the referees will find the work improved as compared to the previous version.

Point-to-point responses are being attached below.

Faithfully,

Hui Yu, Ph. D.


Reviewer's Responses to Questions

**Comments to the Authors:
Please note here if the review is uploaded as an attachment.**

Reviewer #1: Detailed comments:
1. L149-L150: The high GC content of hinges connecting AT-reach tracts (i.e., A/T_A/T or TA_TA) is not surprising. I would say that it is rather expected, as the presence of either A or T in the hinge positions would, in most cases, extended the tracts and would not be counted. The same I think applies to GC-reach tracts that, I expect, are mostly separated by AT-reach hinges.

**Response: Thank you for this insight. We removed all statements regarding elevated GC content. In particular, the relevant supposition in section “Non-canonical RNA-editing events are enriched within MNR adjacency or hinge sites” was rephrased as “*It might be that an actual longer, intact A/T fragment is incorrectly broken into two pieces in the reference genome, which leads to spurious reports of C-to-T RDDs in hinges of A/T MNRs*.”**

2. Table S2: I do not know whether I understand correctly the column ‘Polytract As Microsatellite’. I would expect that the values in the column should be equal (should not be higher) with the values in the ‘Microsatellite’ column. It is the case for most but not for all polytracts. Please explain or correct it.

**Response: Thank you very much for catching a bug of Table S2. Last time when we generated Table S2, we carelessly used an outdated TNR dataset in which some redundancy was present (for example, a string of ABCABCABCA was counted once for the ABC pattern and once for BCA pattern.) We corrected this mistake and modified the table. However, because we provided a detailed table (Appendix S2) for all additional long repeats that are present in the Polytract set yet absent in the UCSC Microsatellites set, we removed original Table S2. The original Table S2 turns into Table 1 in the current response document, in our response to Referee 3. Referring to Table 1 below, you can notice that we used the updated, correct TNR dataset to re-generate the table, where only one intersection column “Concurrent instances” is shown. Now the column “Polytracts” denotes number of unique, non-overlapping polytracts. We also represented the two updated, complex DNR types (CA/GT and CT/GA) in the table this time.**

3. Figure 1E and Figure S2: What is up_down_stream? How it is defined?

**Response: The key words “upstream” and “downstream” were originally defined by ANNOVAR (**[**http://annovar.openbioinformatics.org/en/latest/user-guide/gene/**](http://annovar.openbioinformatics.org/en/latest/user-guide/gene/)**), designating the two 1-kb genomic segments in proximity to the gene body anchored at transcription start/end site. We’ve explained this in particular in caption of Figure 1E.**

4. Maybe I am too pedantic but why the authors sometimes use a lower-case and sometime upper-case letter for different polytracts (e.g., TA or ta) or why sometimes use for DNR e.g., CT and in other places CT/GA. For readers, especially when someone browses the article quickly such inconsistencies may be confusing.

**Response: In Figure 6 which displays the schema of Polytrap, we intentionally used uppercase for MNR and yet lowercase for DNR and TNR. We tried to imply such a distinction – while the motifs of MNR are as is with no ambiguity, the motifs in DNR and TNR account for more patterns than the nominal representation. Say, the MNR “ta” represents both TATATA and ATATAT, and the TNR “aac” accommodates AAC, ACA, CAA, GTT, TGT, and TTG. We intended to indicate this implicit distinction only for Fig.6, because our data release has such uppercase/lowercase distinction. In other places of the main text, we’ve suppressed all unnecessary lowercase representations to reduce confusion as much as possible. Now in Figure 6 (bottom right part) we added a notation to briefly explain the special meaning of lowercase polytract types.**

5. Figure S2 and corresponding text: It is worth to note that GC DNRs are strongly enriched in 5’UTR, exonic, and up_down_stream regions.

**Response: we added this statement in the context – “*The CGG TNR and the GC DNR (Fig. 1F) also have notable over-representation in up/down-stream segments in proximity to gene body and 5’-UTR regions, a phenomenon rarely seen in other polytract subtypes (Figure S2).*”**

6. Does ‘exonic’ mean protein-coding part of exons or exons including UTRs?.

**Response: “exonic” refers to coding exonic portion of a gene body but excluding UTRs, as we have special terms UTR3 and UTR5 to designate 3’-UTR and 5’-UTR segments. We added explanation on UTR3 and UTR5 in caption of Figure 1.**

7. According to Figure 3B (bitmap), CGG TNRs are more similar to AATs that to AACs. The fragment of text commenting Figure 3 should be reconsidered.

**Response: We applied inverse correlation (1-r) as distance metric and employed average linkage to generate the clustering dendrogram. The clustering dendrogram indicated the two subtypes CGG and AAC can be combined before either of them merging with any other leaf nodes. We have to adhere to what the dendrogram reveals. The clustering parameters were added to caption of Figure 3B.**

8. Fragment encompassing L189~L191: Figure 3A does not show repeats “6U or more” but ‘5U or more’ (15+).

**Response: we used to intend “15+” as length>15, which seemed misleading. Now we modified it to “16+” in Figure 3A.**

Reviewer #2: The authors of the revised manuscript “Non-canonical RNA-DNA differences and other human genomic features are enriched within very short tandem repeats” have made substantial improvement over the original manuscript. They have satisfactorily addressed most of the concerns and questions I raised earlier, but I still find the article missing an important component: description of performed statistical analyses. Authors performed various statistical tests as illustrated throughout the manuscript mainly in the Results section and shown in the Figures. Though the fundamental statistical analysis incorporated in the software polytrap is well described in the Materials and methods, a detailed description of the performed ad hoc statistical analyses is missing [including which statistical softwares were used]. In addition, it would be necessary to know for instance based on polytrap output, what is the cut off p-value for statistical significance in enrichment analysis.

**Response:**

**We added a subsection to Materials and Methods under the title “Miscellaneous statistical analyses,” as pasted below.**

**“*All statistical analyses, including the procedures encompassed in software package Polytrap, were conducted in R environment. We employed the hypergeometric probability model to test for the significant enrichment of MNRs/DNRs/TNRs in enhancers (Fig. 2A) based on four key numbers: number of enhancers, number of enhancers embedding polytracts, number of nucleotides in reference genome, and number of nucleotides in reference genome occupied by polytracts. Confidence Interval (95%) of Relative Risk (Fig. 2B) was calculated following the standard way (******45******). In hierarchical clustering analysis (Fig. 3B), distance metric used one minus correlation value and linkage choice was the average method. We employed the over-representation binomial probability model implemented in Polytrap to study RNA-editing events’ enrichment in various polytract species, where the statistical significance was set at nominal p<0.01. Pearson correlation coefficient (r) was calculated and tested for r≠0 when we studied a possible linear correlation relationship between mean length and density of polytracts (Appendix S1: Fig. S1).”***

**For the output of software polytrap, we just specify the p-values and do not assert if they are statistically significant. When the user chooses different sets of polytracts (MNR, MNR+DNR, MNR+DNR+TNR, etc.), the number of Binomial tests that are conducted simultaneously varies. So we do not attempt to apply a fixed cutoff on the reported p-values. In the genomic feature landscape figure, because nearly 100 features were included, we explicitly indicated a threshold line corresponding to p=1e-4. This sentence was added to caption of Figure 5 to explain the choice of 1e04 - “*Because nearly 100 features were tested simulatenously, we applied bonferroni correction and deemed p<1e-4 as statistically significant*.”**

In addition, the figures especially Figures in the main manuscript are not of publishable quality [when you print them you can hardly read the figure annotations and labels]. In addition, in Figure 6, N is the total number of nucleotides in the genome, but it is indicated as if it is the total number of nucleotides excluding the total number of nucleotides occupied by polytracts. By your definition, N in Fig. 6 should be illustrated by blue color + grey color.

**Response: This time we printed out the original figures on A4 paper and confirmed their acceptable resolution. Thank you very much for suggesting to improve Fig. 6 illustration. We’ve modified Fig. 6 to incorporate the specific suggestion.**

Minor typos on lines L412 [I suppose you meant hypergeometric model instead of hypegenometric model], 442-443 [the word “instead” makes the sentence reads a little bit contradictory to the previous sentence].

**Response: Thank you so much for catching this typo on “hypergeometric” for us. It was corrected. As for the word “instead,” we changed it to “as well.”**

Reviewer #3: The article has been reviewed fairly well and most issues have been addressed.

Although an exact tandem repeat finder may hide useful approximate tandem repeats or shorten/break up longer chains, and therefore somehow mislead the statistics you have found, your catalog could help reporting new locations/repeats (absurdly) overlooked before (e.g. in UCSC Simple/Microsatellite Repeats). In this regard, have you checked that your new locations/repeats are correct? Could you justify why they don't appear in the UCSC catalog? Can you confirm that the new locations/repeats are not present in catalogs like the 700k by Willems et al.?

***Response: Thanking you for appreciating our seeking exact tandem repeats. As shown in Table 1 below, our polytract set encompasses all UCSC Microsatellite instances (DNRs and TNRs of 15 or more units), and our set also contains 15-89% additional components that theoretically meet the criterion of UCSC Microsatellites. Now, we identified all additional qualified polytracts (Table 1 below, column “Additional polytracts”) and retrieved their nucleotide sequences using the “getSeq” function from the BioConductor package Biostrings (Appendix S2). Occasionally, these sequences may be one or two bases shorter than the required 15-copy length, because for Polytract the possibly incomplete terminal motif is included and that may falsely upgrade a 14-copy tract to a 15-copy tract. However, the genuine 15-copy or even longer tracts make a significant portion of the dataset. For example, two instances of long (literal) AAC TNR (15+ copies) are included in Polytracts but are missing in UCSC Microsatellites (Figure 1 below). As another example (Figure 2 below), our dataset contains a single qualified segment of chr11:133,140,998-133,141,050, which comprises 17 copies of GAC; this instance of GAC long repeat was missed by UCSC Microsatellites.***

***Please inspect the actual repeat sequences provided in the separate tab-separated file (Appendix S2) to confirm that the additional polytracts do qualify the inclusive criterion of UCSC Microsatellites. In Figure 1 below, we present the UCSC Genome Browser snips for two instances of long AAC tracts (>15 copies), and in Figure 2, we present the UCSC Genome Browser snip for the sole stretch of 17-unit GAC tract that is missed by UCSC Microsatellites. In these UCSC Genome Browser sessions, RepeatMasker, Microsatellites, and Polytracts (the new official Tract Hub contributed by us) were turned on. Microsatellites track was entitled with “Microsatellites – Di-nucleotide and Tri-nucleotide Repeats” and Polytracts track was entitled with “Human MNR/DNR/TNR repeats plus hinges (breakpoints).”***

*Table 1. Numbers of repeats in the UCSC microsatellite set, the Polytract set, and their overlapping set.*

| ***DNR/TNR Repeat category****** | ***Polytracts*** | ***Microsatellites*** | ***Concurrent instances*** | ***Additional polytracts*** | ***Polytract As Microsatellite (%)*** | ***Microsatellite As Polytract (%)*** |
| --- | --- | --- | --- | --- | --- | --- |
| *aac* | *88* | *74* | *74* | *14* | *84%* | *100%* |
| *aag* | *506* | *55* | *55* | *451* | *11%* | *100%* |
| *aat* | *769* | *654* | *654* | *115* | *85%* | *100%* |
| *acc* | *47* | *19* | *19* | *28* | *40%* | *100%* |
| *act* | *35* | *24* | *24* | *11* | *69%* | *100%* |
| *agg* | *43* | *13* | *13* | *30* | *30%* | *100%* |
| *atc* | *67* | *46* | *46* | *21* | *69%* | *100%* |
| *ca/gt* | *41498* | *30816* | *30816* | *10682* | *74%* | *100%* |
| *cag* | *29* | *19* | *19* | *10* | *66%* | *100%* |
| *cgg* | *12* | *7* | *7* | *5* | *58%* | *100%* |
| *ct/ga* | *5165* | *2720* | *2720* | *2445* | *53%* | *100%* |
| *ta* | *11992* | *6891* | *6891* | *5101* | *57%* | *100%* |
| *gac* | *1* | *0* | *0* | *1* | *0%* | *N/A* |
| *gc* | *0* | *0* | *0* | *0* | *N/A* | *N/A* |

**** These refer to long tracts (of 15 or more copies of dimer/trimer motif), because this is the defining criterion of UCSC Microsatellites.***

Figure 1A. the 1^st^ AAC tract that are captured by Polytract set yet missed by UCSC Microsatellites.

Figure 1B. The 2nd AAC tract that are captured by Polytract set yet missed by UCSC Microsatellites.

Figure 2. The sole long repeat of 17 units of GAC motif that is missed by UCSC Microsatellite.

***The 700K catalog proposed by Willems et al. was not publicized. We obtained Table S10 from the publication which listed “Coding STRs with non-reference major alleles.” Table S10 comprised 46 DNRs or TNRs originating from HG19. We confirmed that all 46 repeats compiled by Willems in Table S10 hit at least one polytract from our dataset.***

***Table 2. All 46 repeats from Table S10 of Willems 2014 hit at least one overlapping polytract in our dataset.*** *We just picked one polytract when multiple polytracts overlapped with the query.*

| *Table S10 of Willems et al.* | | | | *Overlapping polytracts* | | | | | |
| --- | --- | --- | --- | --- | --- | --- | --- | --- | --- |
| *chr* | *start* | *end* | *width* | *chr* | *start* | | *end* | *width* | |
| *chr1* | *31905890* | *31905904* | *15* | *chr1* | *31905886* | *31905891* | | | *6* |
| *chr1* | *209605637* | *209605676* | *40* | *chr1* | *209605637* | *209605676* | | | *40* |
| *chr1* | *240255569* | *240255585* | *17* | *chr1* | *240255569* | *240255585* | | | *17* |
| *chr2* | *73613032* | *73613081* | *50* | *chr2* | *73613032* | *73613070* | | | *39* |
| *chr2* | *88926730* | *88926754* | *25* | *chr2* | *88926730* | *88926754* | | | *25* |
| *chr2* | *96780975* | *96780994* | *20* | *chr2* | *96780975* | *96780994* | | | *20* |
| *chr2* | *241696826* | *241696873* | *48* | *chr2* | *241696826* | *241696839* | | | *14* |
| *chr3* | *42251578* | *42251609* | *32* | *chr3* | *42251578* | *42251609* | | | *32* |
| *chr3* | *46751074* | *46751100* | *27* | *chr3* | *46751074* | *46751100* | | | *27* |
| *chr3* | *49395674* | *49395693* | *20* | *chr3* | *49395674* | *49395693* | | | *20* |
| *chr3* | *113376105* | *113376147* | *43* | *chr3* | *113376095* | *113376106* | | | *12* |
| *chr3* | *183493744* | *183493772* | *29* | *chr3* | *183493744* | *183493772* | | | *29* |
| *chr3* | *184429134* | *184429154* | *21* | *chr3* | *184429134* | *184429154* | | | *21* |
| *chr4* | *140651585* | *140651612* | *28* | *chr4* | *140651585* | *140651612* | | | *28* |
| *chr4* | *155244402* | *155244432* | *31* | *chr4* | *155244426* | *155244431* | | | *6* |
| *chr5* | *56177849* | *56177874* | *26* | *chr5* | *56177849* | *56177874* | | | *26* |
| *chr6* | *32191659* | *32191690* | *32* | *chr6* | *32191659* | *32191690* | | | *32* |
| *chr6* | *161519351* | *161519390* | *40* | *chr6* | *161519351* | *161519383* | | | *33* |
| *chr7* | *15725789* | *15725826* | *38* | *chr7* | *15725798* | *15725826* | | | *29* |
| *chr8* | *144511954* | *144511983* | *30* | *chr8* | *144511954* | *144511983* | | | *30* |
| *chr9* | *95237025* | *95237068* | *44* | *chr9* | *95237025* | *95237068* | | | *44* |
| *chr10* | *21805467* | *21805518* | *52* | *chr10* | *21805463* | *21805468* | | | *6* |
| *chr12* | *6777065* | *6777113* | *49* | *chr12* | *6777056* | *6777065* | | | *10* |
| *chr12* | *14720550* | *14720576* | *27* | *chr12* | *14720555* | *14720576* | | | *22* |
| *chr12* | *76424917* | *76424968* | *52* | *chr12* | *76424917* | *76424936* | | | *20* |
| *chr12* | *122359386* | *122359417* | *32* | *chr12* | *122359397* | *122359407* | | | *11* |
| *chr12* | *124887059* | *124887110* | *52* | *chr12* | *124887059* | *124887095* | | | *37* |
| *chr14* | *23744797* | *23744825* | *29* | *chr14* | *23744801* | *23744825* | | | *25* |
| *chr14* | *23744827* | *23744852* | *26* | *chr14* | *23744827* | *23744846* | | | *20* |
| *chr14* | *93154538* | *93154554* | *17* | *chr14* | *93154538* | *93154554* | | | *17* |
| *chr14* | *105996050* | *105996069* | *20* | *chr14* | *105996050* | *105996069* | | | *20* |
| *chr15* | *78913058* | *78913089* | *32* | *chr15* | *78913068* | *78913089* | | | *22* |
| *chr17* | *6928020* | *6928049* | *30* | *chr17* | *6928020* | *6928049* | | | *30* |
| *chr17* | *7750167* | *7750216* | *50* | *chr17* | *7750167* | *7750175* | | | *9* |
| *chr17* | *17039562* | *17039596* | *35* | *chr17* | *17039562* | *17039596* | | | *35* |
| *chr17* | *17697094* | *17697134* | *41* | *chr17* | *17697094* | *17697134* | | | *41* |
| *chr18* | *48723138* | *48723163* | *26* | *chr18* | *48723138* | *48723153* | | | *16* |
| *chr18* | *72223582* | *72223608* | *27* | *chr18* | *72223592* | *72223608* | | | *17* |
| *chr19* | *11558341* | *11558405* | *65* | *chr19* | *11558341* | *11558372* | | | *32* |
| *chr19* | *17397457* | *17397522* | *66* | *chr19* | *17397457* | *17397500* | | | *44* |
| *chr19* | *56599438* | *56599454* | *17* | *chr19* | *56599453* | *56599464* | | | *12* |
| *chr20* | *278688* | *278703* | *16* | *chr20* | *278688* | *278703* | | | *16* |
| *chr20* | *44520238* | *44520263* | *26* | *chr20* | *44520238* | *44520263* | | | *26* |
| *chrX* | *57618726* | *57618749* | *24* | *chrX* | *57618726* | *57618749* | | | *24* |
| *chrX* | *119387834* | *119387853* | *20* | *chrX* | *119387834* | *119387853* | | | *20* |
| *chrX* | *120009227* | *120009252* | *26* | *chrX* | *120009232* | *120009245* | | | *14* |

In addition, I would strongly suggest to emphasize earlier, rather than in the Discussion section, that the main differences between DNA and RNA found within or adjacent to monomer repeats may have been caused by artifacts (e.g. sequencing errors, alignment consent errors) or potential (low-frequency) polymorphisms, and similarly for what is reported for somatic mutations in cancer genomes for both mononucleotides and trinucleotides (i.e. the size of codons).

**Response: we added these statements in Introduction –**

**“*As we reviewed earlier (******15******), RDDs observed in RNA-Seq data comprise real RNA-editing events, single-nucleotide polymorphisms, and artifacts (errors). Actually, there is a strong voice that non-canonical RNA editing events are primarily attributed to sequencing errors or bioinformatics pitfalls (******16******). Very short tandem repeats, such as MNRs and DNRs, pose higher sequencing/analysis challenges than other genomic regions, so RDDs around very short tandem repeats may be especially suspicious*.”**

Reviewer #4: The authors have almost answered questions concerned by me. They added related information in methods part, and rewrited the disscussion. The new version looks much better than last version. I suggest to accept the manuscript to publish on the Plos Computaional Biology.

**Response: Thank you very much!**

**Have all data underlying the figures and results presented in the manuscript been provided?**
Large-scale datasets should be made available via a public repository as described in the *PLOS Computational Biology* [data availability policy](http://journals.plos.org/ploscompbiol/s/data-availability), and numerical data that underlies graphs or summary statistics should be provided in spreadsheet form as supporting information.

Reviewer #1: Yes

Reviewer #2: Yes

Reviewer #3: Yes

Reviewer #4: Yes
